# RNA Cleavage Properties of Nucleobase-Specific RNase MC1 and Cusativin Are Determined by the Dinucleotide-Binding Interactions in the Enzyme-Active Site

**DOI:** 10.3390/ijms23137021

**Published:** 2022-06-24

**Authors:** Priti Thakur, Jowad Atway, Patrick A. Limbach, Balasubrahmanyam Addepalli

**Affiliations:** Rieveschl Laboratories for Mass Spectrometry, Department of Chemistry, University of Cincinnati, Cincinnati, OH 45221, USA; thakurpi@mail.uc.edu (P.T.); Jowad1998@gmail.com (J.A.); limbacpa@ucmail.uc.edu (P.A.L.)

**Keywords:** ribonucleases, MC1, cusativin, LC-MS, cleavage specificity, cleavage efficiency, dinucleotide-specific

## Abstract

Knowledge of the cleavage specificity of ribonucleases is critical for their application in RNA modification mapping or RNA-protein binding studies. Here, we detail the cleavage specificity and efficiency of ribonuclease MC1 and cusativin using a customized RNA sequence that contained all dinucleotide combinations and homopolymer sequences. The sequencing of the oligonucleotide digestion products by a semi-quantitative liquid chromatography coupled with mass spectrometry (LC-MS) analysis documented as little as 0.5–1% cleavage levels for a given dinucleotide sequence combination. While RNase MC1 efficiently cleaved the [A/U/C]pU dinucleotide bond, no cleavage was observed for the GpU bond. Similarly, cusativin efficiently cleaved Cp[U/A/G] dinucleotide combinations along with UpA and [A/U]pU, suggesting a broader specificity of dinucleotide preferences. The molecular interactions between the substrate and active site as determined by the dinucleotide docking studies of protein models offered additional evidence and support for the observed substrate specificity. Targeted alteration of the key amino acid residues in the nucleotide-binding site confirms the utility of this *in silico* approach for the identification of key interactions. Taken together, the use of bioanalytical and computational approaches, involving LC-MS and ligand docking of tertiary structural models, can form a powerful combination to help explain the RNA cleavage behavior of RNases.

## 1. Introduction

Ribonucleases (RNases) play critical roles in gene expression, RNA maturation, quality control, and microbial defense [1]. These RNA hydrolyzing enzymes can be endonucleases (cleave internal phosphodiester bonds) or exonucleases (hydrolyzing from 5′ or 3′ termini) [1]. Endoribonucleases are grouped into RNase A, T1, and T2 families based on structure, pH optima, and nucleobase preference [2,3]. Nucleobase preference for RNase A (pyrimidine-specific) [4,5,6] and RNase T1 (guanosine-specific) [7] is primarily determined by the B1 or 5′ base binding site with no strict preference in the B2 site under typical digestion conditions [8].

The RNase T2 family members—MC1 [9] and cusativin [10]—exhibit novel RNA cleavage features. RNase MC1 cleaves RNA at the 5′ end of uridine [11] and cusativin at the 3′ end of cytidine [12]. Simple atomic substitutions (C=O to C=S) or methylations make the modified uridine a non-substrate for MC1 [11]. The influence of chemical modifications on the cleavage property of these enzymes seems to depend on the altered chemical groups and interactions in the active site of protein tertiary structure [11,12,13,14,15,16]. While methylation of cytosine (m^5^C) does not affect its recognition as a substrate, RNA with tandem cytidines is not a substrate for cusativin [12], indicating differences in the binding site architecture (B1 and B2 sites) of these enzymes. Hydrogen bonding interactions with Gln9, Asn71, and the hydrophobic sandwich of uracil between Leu73 and Phe80 in the B2 site are reported to be responsible for the uridine specificity of MC1 [9]. Recently, altered specificity of these enzymes was reported based on the type of heterologous host used for protein expression [17]. These and our own studies [15] indicate that specific dinucleotide bonds (GpU for MC1 and CpC for cusativin) are not cleaved even though the appropriate nucleoside (either uridine or cytidine) is present in the sequence. Therefore, the existing information does not fully explain the cleavage preferences of these enzymes, and more systematic efforts are warranted to understand the RNA cleavage behavior.

Nucleobase specificity of RNases is computed by the spectrophotometric kinetic analysis using dinucleotide or homopolymer substrates [17,18] and from the structural analysis of enzyme-nucleotide complexes [9,19,20]. Tertiary structures predicted based on the evolutionarily related structural templates of homologous proteins [21,22] are also being used for drug discovery, design, and understanding the site-specific interactions with small molecules or proteins [23,24]. Studies involving such tertiary structures and dinucleotide sequences could also help explain why bonds in certain dinucleotide sequences are not cleaved by MC1 and cusativin despite having the appropriate nucleotide in a sequence. Detailed understanding of the cleavage characteristics of ribonucleases would also be helpful for effective characterization of the mRNA vaccine analytes or RNA therapeutics [25] apart from the cellular epitranscriptome (post-transcriptional modifications in RNA). Reprogramming of these modifications in response to physiological changes including stress and pharmaceuticals has been documented [26].

Here, we demonstrate the dinucleotide-specific cleavage behavior of MC1 and cusativin through semi-quantitative LC-MS/MS analysis of oligonucleotide digestion products from a customized RNA sequence. This data is further supported by the in silico molecular docking of dinucleotide ligands to the enzyme active site, where the identified interactions between the nucleobases and key amino acid residues provide a molecular explanation for the observed RNA cleavage. Alteration of representative amino acid residues in the active site predictably altered the cleavage properties of the enzyme, indicating the utility of these approaches for understanding the cleavage specificity and efficiency of nucleobase-specific ribonucleases.

## 2. Results

### 2.1. Dinucleotide-Based Substrate Specificity and Cleavage Efficiency of RNase MC1 and Cusativin

For a comprehensive understanding of the cleavage characteristics of ribonucleases, we designed a 31-nt RNA (GCAUCAGAAAUACACCCGUAGGGCUUUGAGA) containing all dinucleotide combinations, including homopolymers. We analyzed the enzymatic digestion products by liquid chromatography coupled tandem mass spectrometry (LC-MS/MS) in a semi-quantitative fashion using RNA ModMapper to reveal the cleavage specificity and efficiency. To confirm the applicability of this approach, we first analyzed RNase T1 digestion products. We observed cleavage of all the GpN (N is any nucleoside) bonds with no undercutting or missed cleavages (Supplemental Figure S1) under optimal digestion conditions.

#### 2.1.1. RNase MC1

**Figure 1:**
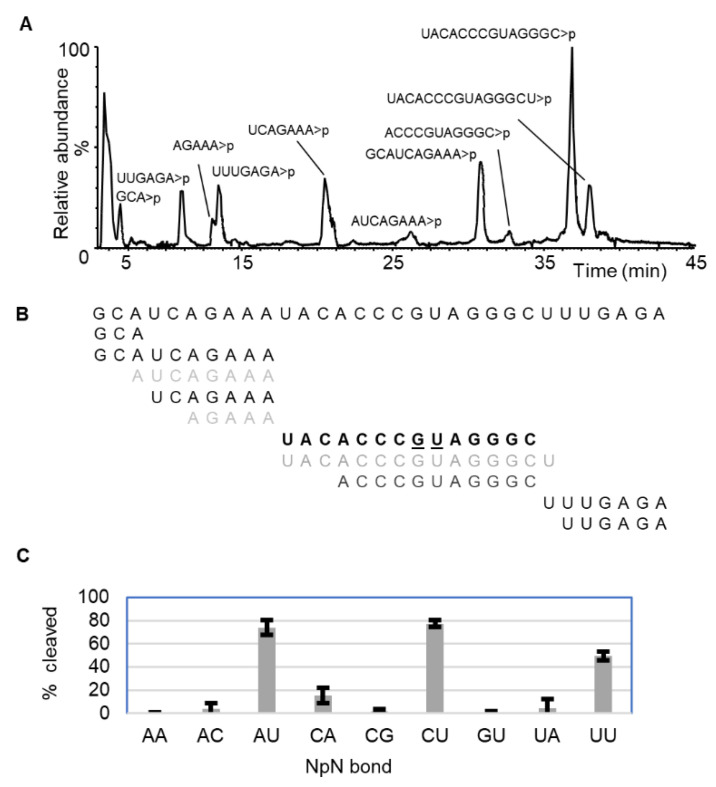
Dinucleotide sequence-dependent RNA cleavage by RNase MC1. (**A**) Total ion chromatogram (TIC) annotated with the observed digestion products containing cyclic phosphates (>*p*). (**B**) Overlay of the observed digestion products mapped to the original sequence. (**C**) Dinucleotide bonds cleaved by the MC1. Cleavage efficiency is the percentage of abundance (peak area) ratio of the expected/observed digestion product upon cleavage of specific dinucleotide and that of the undercut sequences overlapping respective bonds.

Incubation of the 31-nt RNA with MC1 revealed a characteristic cleavage at the 5′ end of uridine at ApU (70–100%), CpU (80–100%), and UpU (45–60%) bonds (Figure 1, Table 1). However, despite having uridine at a 3′ position (of dinucleotide) cleavage was negligible for the GpU bond (1.0–1.5%). Surprisingly, the CpA bond also exhibited moderate cleavage efficiency (10–20%), while the other bonds, such as ApA, ApC, CpG, and UpA, were cleaved at extremely low efficiency (0–5%), suggesting that specific dinucleotide combinations are preferred for RNA cleavage. Analysis of MC1 digestion products of yeast tRNA^Phe^ revealed identical cleavage behavior with unmodified sequences. Cleavages involving modified nucleotides exhibited interesting behavior. The bonds for dihydrouridines ([D]p[D]) and [m^5^C]pU are cleaved with high efficiency (64–68% and 99–100%, respectively) (Supplemental Figure S2) suggesting that the dihydrouridine is indistinguishable from uridine as a substrate. However, Gp[D], [m^7^G]pU, and [m^1^A]pU bonds were not cleaved indicating that guanosine or methylated adenine at the 5′ positions (binds to the B1 region of enzyme active site) of dinucleotide makes it a non-substrate (Supplemental Figure S2).

#### 2.1.2. RNase Cusativin

**Figure 2:**
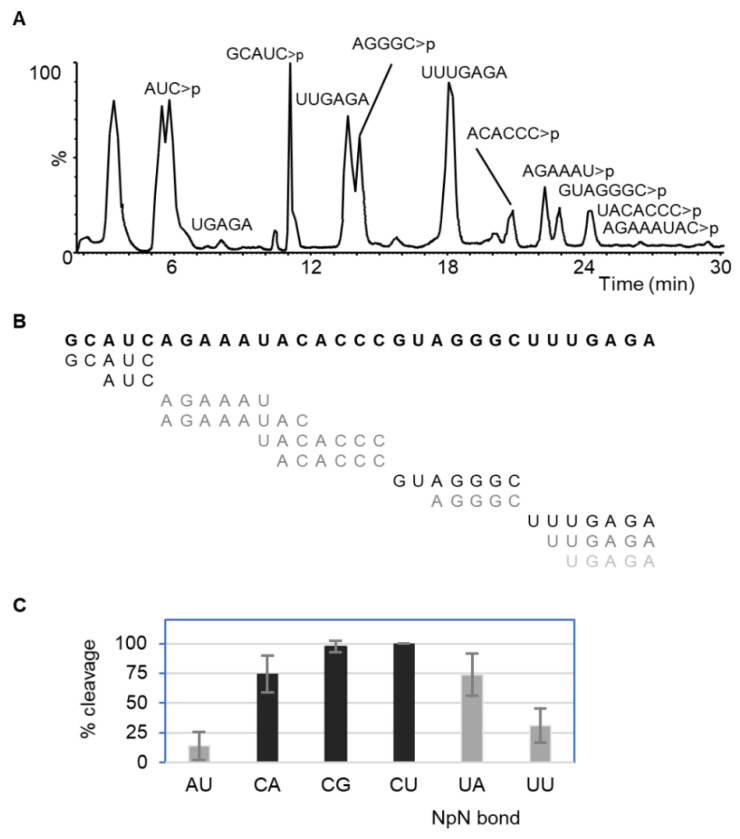
Dinucleotide sequence-dependent RNA cleavage by RNase cusativin. (**A**) TIC annotated with the observed digestion products containing cyclic phosphates (>*p*). (**B**) Overlay of the observed digestion products mapped to the original sequence. (**C**) Dinucleotide bonds cleaved by the cusativin. Cleavage efficiency is the percentage of abundance (peak area) ratio of the expected/observed digestion product upon cleavage of specific dinucleotide and that of the undercut oligonucleotide sequences overlapping respective bonds.

Incubation of the 31-mer with cusativin resulted in efficient cleavage at the 3′ ends of cytidine at CpG, CpU (80–100%), CpA bonds as well as UpA (70–90%) (Figure 2, Table 1). This data implies that RNA cleavage is manifested if the 5′ base is cytosine, or uracil if the 3′ base is adenine (in the dinucleotide UpA). However, cleavage does not happen if the 3′ base is cytosine, because no cleavage of CpC, UpC, GpC, ApC bonds was noticed. Interestingly, moderate cleavage (14–30%) was also observed for ApU and UpU bonds. Taken together, RNA cleavage is allowed for adenine, guanine, and uracil, potentially through recognition at the 3′ base position, but the 5′ base recognition depends on the dinucleotide combination similar to MC1 (Table 1). Analysis of the digestion products of yeast tRNA^phe^ revealed additional cleavage for Cp[m^2,2^G] (48–62%) and [m^5^C]pU (98–100%) bonds, suggesting that methylation in any position of the dinucleotide is not a hindrance to the cusativin-mediated RNA cleavage (Supplemental Figure S3).

### 2.2. In Silico Molecular Docking of RNase Active Site with Nucleotide Substrates

To understand any correlation between the RNA cleavage behavior and the molecular docking scores of ligands bound to the active site, RNase T1 and A models were evaluated initially.

#### 2.2.1. Docking RNase T1 with Single Nucleotide Ligands

A tertiary structure of RNase T1 (GenBank: RMZ48028.1) was generated by homology modeling and docked with a 3′-GMP ligand. This docked ligand exhibited identical overlay with the 3′-GMP bound T1 crystal structures—4GSP [27] or 1RGA [28] (Supplemental Figure S4) with high fitness score or solution validation parameters including PLP (Piecewise Linear Potential: 72), Gibbs free energy change (−20 kcal/mole) and high number (14) of hydrogen bonding interactions.

Docking with dinucleotides, GpU, CpU, and ApU revealed interesting observations (Supplemental Figure S5). The docked model of T1 with GpU exhibited 16 hydrogen bonds (guanine—7, ribose phosphate—7, and uracil—2) and a suitable fitness score (PLP: >99 and ΔG: −18 kcal/mole). Guanosine binding is mirrored accurately with the GpU bound T1 crystal structure—1B2M [29] drawing hydrogen bonding interactions between guanine and Asn98, Glu46, Tyr45, Asn44, Asn43, Tyr42 on one hand, ribose phosphate and His40, His90, Glu58, Arg77, Tyr38, Asn36, on the other. The only difference is that the uridine was oriented away from the polypeptide in the crystal structure, while the docked solution showed a hydrogen bond with Ser35 and Asn36.

Docking with a non-substrate, CpU, yielded a low fitness score (PLP: 60.6 and ∆G: −5.4 kcal/mole). Cytosine formed only two hydrogen bonds with Glu46 and Asn98 and the orientation of 3′ uracil was altered. Similarly, docking T1 with ApU revealed the formation of two hydrogen bonds between adenine and Asn43 and Glu46 as opposed to seven bonds for guanine. However, the phosphate group was positioned correctly in the catalytic site, making H-bonds with appropriate amino acid residues, Asn36, Tyr38, and Arg77 (overall PLP: 80.08 and ∆G: −7.33 kcal/mole). Thus, fewer hydrogen bonds with the 5′ base in the active site could be the reason for negligible or no cleavage of CpU or ApU bonds (Supplemental Table S1) under optimal cleavage conditions (2 h), even though these dinucleotides may be classified as valid targets based on the high fitness (PLP > 50) and ChemScore (ΔG) by GOLD software [30,31]. Incidentally, an extended period of incubation (>16 h) with RNase T1 leads to cleavage of poly(A) tails in RNA [32].

#### 2.2.2. Docking RNase A with Dinucleotide Ligands

A homology model of RNase A (GenBank: AAB35594.1) was generated and docked with dinucleotide ligands. The docked model with CpA exhibited a high fitness score (PLP: 74.8, ∆G: −16.10 kcal/mol) and 12 hydrogen bonds (cytosine with Thr45, Asp83, Ser123, Phe120, ribose phosphate with Lys41, His12, His119, Gln11, Asp121, Lys7, and adenine with Asn67 and Gln79) in the binding site (Supplemental Figure S6). This docked solution aligned well with the CpA-bound RNase A (PDB ID 1RPG) crystal structure [33]. Docking with UpA yielded a similar trend of fitness scores (PLP: 74.2, ΔG: −8.10 kcal/mol, hydrogen bonds (uracil with Thr45, Ser123, Phe120, ribose phosphate with Lys41, His12, His119, Gln11, Asp121, and adenine with Asn67, Glu111, and Gln79)). However, the GpA ligand bound to the active site in a 3′–5′ direction with a low fitness score (PLP: 59.5, ΔG: 1.5 kcal/mol, and 6 hydrogen bonds (adenine with Thr45, Phe120, ribose phosphate with Lys41, His119, Gln11, and guanine with Glu111)). Decreased number of hydrogen bonds in the catalytic site (with ribose phosphate) coupled with the increased free energy of binding (Supplemental Table S2) could be responsible for the lack of cleavage for GpA. Thus, molecular docking studies involving predicted tertiary structures (by homology modeling) and dinucleotide ligands recapitulate the ligand-binding interactions of crystal structures and help explain the cleavage behavior.

#### 2.2.3. Docking RNase MC1 with Nucleotide Ligands

**Figure 3:**
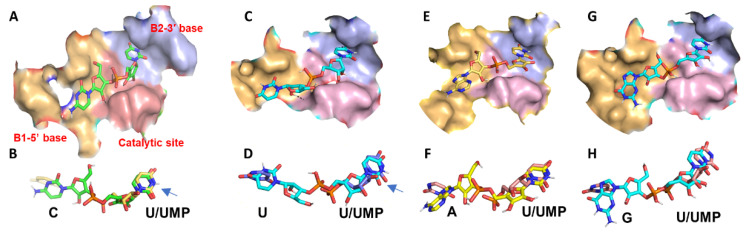
Interactions of RNase MC1 with different dinucleotides. (**A**) Surface view of RNase MC1 docked with CpU dinucleotide. (**B**) Overlay of docked CpU (green) and the bound 5′-UMP in crystal structure, 1UCD (yellow). (**C**) Surface view of RNase MC1 docked with UpU. (**D**) Overlay of docked UpU (cyan) and 5′-UMP of 1UCD (yellow). (**E**) Surface view of RNase MC1 docked with ApU. (**F**) overlay of the docked ApU (light yellow) and 5′-UMP of 1UCD (orange) (**G**) surface view of RNase MC1 docked with GpU, (**H**) Overlay of docked GpU (cyan) and 5′-UMP of 1UCD (orange). The surface view with base recognition and catalytic sites (depicted in different colors) was exported following the ligand docking to MC1 by docking software GOLD.

When a homology protein model generated with the MC1 amino acid sequence (GenBank—AKU36755.1) was docked with 5′-UMP, it recapitulated a precise match with the 5′-UMP bound B2 site of the MC1 crystal structure (PDB: 1UCD) (Supplemental Figure S7). Docking with NpU dinucleotides (where N is C/U/A/G) yielded additional insight into the recognition features at B1 and catalytic sites as well (Figure 3, Supplemental Tables S3 and S4), which were not illustrated before.

##### Docking MC1 with CpU

With the CpU ligand, uridine docked well into the B2 site, making hydrogen bonds with Gln9 (O4), Asn71 (O2 and N3), and Val72 (O4). Further, Leu73 along with Phe80 made π-π interactions identical to 1UCA, 1UCC, and 1 UCD crystal structures [9] (Supplemental Figure S8). The water molecule that mediated interaction between Gln164 and Gln9 in the crystal structure [9] was represented by the arrangement of their side chains (3.4 Å apart) in the docking solution. Although the catalytic residues, His88 and His34, were located a bit far (4.3 Å and 5 Å, respectively) away from phosphate, His88 made a hydrogen bond with 2′-OH of 5′-ribose, while His83 and Glu84 stabilized the 3′-ribose through H-bond interactions at 2′-OH. Lys87 is associated with both B2 and B1 sites through H-bonding with phosphate, 2′-OH, and 3′-OH of the 5′ and 3′ ribose, respectively. Other residues involved with B1 site recognition include Arg150, Gln164, Trp37, and Ser44. Arg150 formed two hydrogen bonds with the oxygen at the C2 position of cytosine and the ring oxygen of ribose. The oxygen in the 5′-CH_2_-O-P- is stabilized by Arg150 and Gln164 with hydrogen bonds. Trp37 aligned below the base and ribose in the B1 site to form π-π interactions. Arg181(3.2 Å), Ser44(3.6 Å), and Thr43(3.6 Å) showed weak hydrogen bonds with N4 of cytosine (Supplemental Figure S8A).

##### Docking MC1 with UpU

Uridines in the UpU made interactions similar to CpU except that the 5′ U made a small turn (1 Å) to the right orienting it slightly away compared to the cytosine in the CpU ligand (Supplemental Figure S8B). However, this did not disrupt the hydrogen bonds with the catalytic site residues (Lys87, Glu84, His83, His88, and His34). Although the orientation of 5′ phosphates is different, it is still H-bonded with Lys87 in the catalytic site. Further, unlike Ser44 making H-bond interactions with cytosine for CpU, no such interaction was noticed for 5′-uracil in the UpU ligand. However, O4 of 5′ uracil made two H-bonds with Arg181 suggesting a stabilization for cleavage.

##### Docking MC1 with ApU

ApU also docked to the active site of MC1 similar to the CpU ligand. The amino group and N7 of adenine made hydrogen bonds with Thr43 and Ser44 in the B1 site, facilitating its stabilization (Supplemental Figure S9A), unlike the cytidine in CpU.

##### Docking MC1 with GpU

The docked solution showed 5′ guanosine binding to the B2 site in 8 of the 10 poses of each docking operation (80% of the time), while phosphate and uracil did not bind to the respective catalytic and binding sites. When bound to the B1 site, guanine showed hydrogen bonding with Arg181, Thr43, Gly42, and Ser44. These interactions, however, stretched the 2′-OH of the 5′ nucleoside away (6.5 Å) from the catalytic site leading to the formation of only one hydrogen bond with Lys87. In the B2 site, only O4 of uracil interacted with Gln9. No bonds were observed with Val72 and Asn71. Further, π-π interaction with Phe80 was weak due to the distance constraints (1 Å away) in the B2 site. Thus, strong interactions in the B1 site and weaker interactions in the B2 site seem to orient the phosphodiester bond away from the catalytic site. Alignment of CpU and GpU docked solutions also showed significant differences in orientation (Supplemental Figure S9B). All these features help explain the inability of the enzyme to cleave the GpU bond even though uridine is present at an appropriate position. Thus, the docking studies of homology models not only recapitulated the single nucleotide binding orientation reported in the crystal models but also provided a rationale for molecular interactions with the dinucleotide ligands.

### 2.3. Homology Modeling of Cusativin and Docking with Dinucleotide Ligands

To help explain the RNA cleavage behavior of cusativin, its tertiary structure was generated through homology modeling and used to dock the dinucleotide ligands. To enable this, BlastP analysis of the cusativin amino acid sequence (GenBank: AAB32233.1) was performed to find a list of similar RNase proteins. Of these, MC1 and cusativin exhibited 53% identity and 69% overall similarity in their amino acid sequences (Supplemental Table S5 and Figure S10). Heterologous expression of MC1 and cusativin in *E. coli* also yielded comparable-sized fusion proteins. Further, homology modeling of the cusativin sequence yielded a similar list of template 3D structures (Supplemental Table S6), and the predicted model structure aligned well with the RNase MC1 crystal structure (PDB: 1BK7) [34]. This model exhibited mismatches in the loop regions corresponding to amino acid residues—I44–47, 181–183, and 205–208 (Supplemental Figure S10B). This model was used for subsequent docking investigations with dinucleotide ligands in the absence of crystal models.

#### 2.3.1. Docking Cusativin with CpU

**Figure 4:**
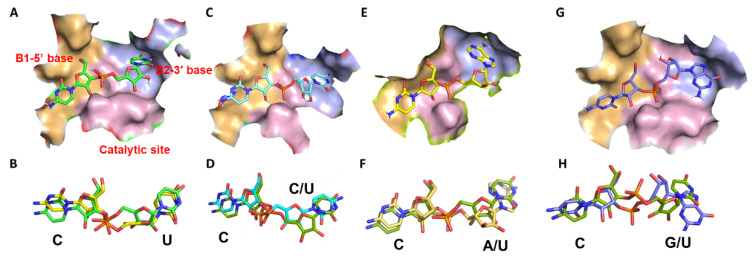
Surface view of the active site of cusativin homology model structure bound with dinucleotide ligands. (**A**) Cusativin docked with CpU, (**B**) Overlay of the CpU (green) docked to cusativin and the CpU docked to the MC1 (yellow) (**C**) Cusativin docked with CpC, (**D**) Overlay of the docked CpC (cyan) and CpU (green) bound to cusativin, (**E**) Cusativin docked with CpA dinucleotide, (**F**) Overlay of docked CpA (yellow) and CpU (green), (**G**) Cusativin docked with CpG (blue), (**H**) Overlay of docked CpG (blue) and CpU (green).

Seven different dinucleotide combinations (CpN (where N is U/A/G/C), UpA, UpU, and ApU) were evaluated for their binding interactions in the cusativin active site based on the RNA cleavage patterns of the enzyme (see Figure 2). A detailed view of ligand binding to the active site is shown in Figure 4 and the hydrogen bonding interactions in Supplemental Tables S7 and S8. Docking with the CpU ligand showed uracil binding to the B2 site through hydrogen bonds with Gln36 (O4), Asn97 (O2 and N3), Val98 (O4), and hydrophobic interaction with Val99 and Phe106 (Supplemental Figure S11A). The ribose portion of uridine exhibited only one hydrogen bond between its 3′-OH and His109. On the other hand, the ribose-3′- phosphate that linked the cytosine and uracil exhibited four hydrogen bonds in the catalytic site involving His114 (and 3′-OH), His60, and Lys113. The ribose and cytosine in the B1 site formed stacking interactions with Trp63 and exhibited hydrogen bonding between Arg176, Ser70, and O2, N4 of cytosine. Arg176 made hydrogen bonds with the ribose ring oxygen and 5′-CH_2_OH of cytidine in the B1 site. Lys178 and Glu189 stabilized the 5′ phosphate. Thus, an extensive network of hydrogen bonding interactions facilitates efficient recognition and cleavage.

#### 2.3.2. Docking Cusativin with CpA

The interactions of cytidine in CpA are identical to those of CpU, where the amino acid residues, His60, 114, 109, Glu110, and Lys113 in the catalytic site made H-bonds. However, adenine exhibited slightly weaker hydrophobic interactions with Phe106 and Val99 (3.8 Å separation distance compared to 3.5 Å for CpU) and no interaction with Val98 in the B2 site. Adenine formed two hydrogen bonds with Gln36 (N1) and Asn97 (N6) (Supplemental Figure S11B). Both CpA and CpU exhibited a similar overlay (Supplemental Figure S11C), indicating efficient recognition and cleavage.

#### 2.3.3. Docking Cusativin with CpC

The 5′-cytosine in the CpC bound to the B1 site similar to CpU. However, binding at B2 was different as cytosine did not show interactions with Val98 and Gln36. The bonding of Asn97 to N4 of cytosine made the base position slightly toward the right side. Moreover, incorrect positioning of Val99 led to the absence of hydrophobic interactions with 3′ cytosine. Further, the CpC ligand exhibited a stretched-out form in the active site, unlike the other dinucleotides, which were in a bent form. These alterations could collectively be responsible for the lack of cleavage for this bond (Supplemental Figure S12).

#### 2.3.4. Docking Cusativin with CpG

The binding of cytosine to the B1 site was identical to that of the CpU ligand. The phosphate group made bonds with Lys113, Glu110, and His60. Binding in the B2 site, however, showed variations. They include the formation of an H-bond between 2′-OH of ribose and the side-chain amide nitrogen of Gln36. The guanine ring was positioned to allow π-π and hydrophobic interactions with Phe106 and Val99. Further, Asn97 formed a hydrogen bond with N7 and O6 of the purine ring, stabilizing its position in the B2 site. Additionally, Thr100 formed a hydrogen bond with the carbonyl group of guanine (Supplemental Figure S13). These interactions were sufficient for efficient recognition and cleavage.

#### 2.3.5. Docking Cusativin with UpA, ApU, and UpU Ligands

Cusativin also exhibited a significant level of cleavage at UpA, ApU, and UpU bonds, making the case for investigating the docking interactions. In 50% of the docking poses, uracil in UpA is bound to the B2 site, and no cleavage could be expected because of the incorrect positioning. In the other 50% of cases, uracil bound to the B1 site with the appropriate number of H-bonds, and it aligned well with CpU. Thus, a good overlap between pyrimidine bases, 5′ ribose, and phosphate was noted in this docking solution. At the B2 site, the purine ring was bonded to a new amino acid, Pro96, along with the Gln36 and Asn97 (Supplemental Figure S14), enabling stabilization for cleavage. ApU, on the contrary, showed different behavior at the B1 site. The adenine formed hydrogen bonds with Arg176 for the pyrimidine portion and Ser70 for the imidazole portion. However, the purine ring was placed at a prohibitive distance to make π-π interaction with Trp63. Although hydrogen bonds were noticed with Lys113 in the catalytic site, the ribose phosphate was positioned at a slightly longer distance (5 Å) from the putative catalytic residue His114 (Supplemental Figure S15). Such a placement could be responsible for lower levels of cleavage for ApU. The UpU ligand occupied the enzyme active site in the same fashion as CpU, indicating that cleavage could be possible. The only difference was that Lys113 made H-bond with O2 of the 5′ base instead of ribose. Further, 2′-OH pointed upward, thereby, potentially reducing the interactions with the catalytic site residues (Supplemental Figure S16) and lower levels of cleavage for UpU.

### 2.4. Effects of the Site-Directed Mutagenesis in the Cusativin-Active Site

To confirm the correlation of docking solutions to the observed cleavage behavior of cusativin, we examined the effects of alterations of Asn97 or Thr100 on the cleavage characteristics of cusativin. Asn97 in cusativin (identical to the Asn71 in MC1) could play a significant role in the stable binding of the 3′ nucleobase in the dinucleotide substrates—CpU, CpA, CpG, UpA, and CpC in the B2 site, although such binding tilted CpC slightly to the right (see above). Alteration of Asn97 to Ser97 led to altered RNA cleavage characteristics for the mutant protein. The cleavage activity was reduced at 60 ^o^C compared to the wild-type protein; however, the mutant exhibited additional cleavages at GpA (6–10%), GpC (10–20%), and GpU (11–15%) in a reproducible fashion. Cleavages also occurred at ApA, ApG, CpC, GpG, and UpG bonds but at an extremely low level (2–5%). Such cleavages were not existent for the wild-type protein (Supplemental Figure S17A). This evidence confirms the requirement of Asn97 for the nucleobase specificity of cusativin, whose influence is similar to that of MC1 (Asn71 as per MC2 numbering) [35].

Thr100 is associated with the recognition of guanosine in CpG dinucleotide and its corresponding position in MC1 contains Arg. Alteration of Thr100 to Arg100 resulted in additional RNA cleavages at GpA (8–9%) and GpC (10–12%). Cleavages at ApC, ApG, and CpC were also observed at about 1–3% by this protein (Supplemental Figure S17B). These observations suggest that the mutation of key amino acid residues in the B2 site could also influence the nucleotide recognition in the B1 site, thereby altering the dinucleotide-dependent RNA cleavage characteristics.

#### 2.4.1. Enzyme Kinetic Analysis of Mutants

Steady-state enzyme kinetic analysis (Supplemental Figure S18 and Table S9) of the mutants revealed significant deviations compared to the wild type. While T100R exhibited higher Km (34.7 vs. 21.8 μM), N97S showed a lower value (18.6 μM). However, T100R exhibited a higher Vmax (0.47 vs. 0.32 μM min^−1^), N97S showed a decreased value (0.187 μM min^−1^), and a similar trend was observed for Kcat (0.74, 1.08, and 0.43 min^−1^ for WT, T100R, and N97S, respectively), indicating that the catalytic activity is also affected apart from the substrate specificity.

#### 2.4.2. Docking Interactions of Cusativin Mutants

The altered specificity of the mutants is further probed by docking the mutant protein structures with the newly observed dinucleotide ligands. Figure S19 shows the docking scores of GpA, GpC, and GpU ligands with the N97S mutant. In all these cases, the serine substitution exhibited two additional H-bonding interactions with the 3′ base (A or C or U). Although GpC exhibited a lower number of hydrogen bonds (7 vs. 10 or 12 for GpA and GpU, respectively), a slightly higher level of cleavage (10–20% compared to 6–10% for GpA) for this dinucleotide is interesting. Nevertheless, serine substitution alters the cleavage preference besides causing a reduction in activity.

T100R substitution led to a switch of hydrogen bonding interactions from the nucleobase (Supplemental Figure S13) to ribose (Supplemental Figure S20). This could pull the ligand more toward that B2 site to facilitate the interactions of the phosphodiester bond with the catalytic site. However, such an interaction was not observed with adenosine in the GpA ligand, keeping the ligand away from the catalytic region. Thus, substitutions in the B2 site seem to adjust the active site in such a way that guanosine is accommodated to cleave the GpC, GpA, or GpU bond. These studies, nevertheless, reinforce the dinucleotide-specific cleavage behavior of cusativin.

## 3. Discussion

The traditional way of determining the substrate specificity of ribonucleases through activity assays using spectrophotometry or denaturing gels [5,17,18] requires a minimum of 16 different dinucleotide combinations in a sequence when the enzyme specificity is unknown. The use of a single RNA substrate with 16 dinucleotide combinations and homopolymer sequences is a viable alternative to understanding RNase cleavage preferences. Characterization of the oligonucleotide digestion products by LC-MS, which is a semi-quantitative technique, could provide a readout of the ribonuclease specificity as each digestion product is a result of two endonucleolytic cuts—one at the 5′-end and the other at the 3′end. Computing the relative abundance of a target digestion product compared to those with the overlapping regions at 5′ and 3′ ends can reveal the cleavage efficiency. One limitation or requirement of this approach is the complete sequencing of the oligonucleotide digestion products following enzymatic treatment. The use of a known sequence and LC-MS/MS-based analysis can fulfill such requirements. Such a strategy in combination with docking dinucleotide ligands to the enzyme active site can offer a molecular explanation for the observed cleavage features. Studies with RNase T1 (Supplemental Figures S1, S4 and S5) and RNase A (Supplemental Figure S6) homology models have supported this hypothesis and its applicability. This strategy is successful with high-quality intact RNA and high-purity protein samples. However, the precise bias of the enzyme may not be perfectly represented in all cases, especially if the enzyme lacks nucleobase preference. This strategy may also not work for sequence-specific endoribonucleases such as MazF and its homologs, which recognize longer (3–6 nt) sequences [36,37].

### 3.1. Cleavage Specificity and Efficiency of RNase MC1

Structural models reveal the optimal interactions required for recognition and catalysis when the substrate binds to the active site. Crystal structures of 2′ UMP or 3′ UMP bound MC1 revealed the role of Gln9, Asn71, Leu73, and Phe80 for uridine-specific binding in the B2 site [9,38], which explains the efficient cleavage of ApU, CpU, and UpU bonds, but not GpU. The use of a customized RNA sequence in combination with LC-MS could document the cleavage percentage as little as ~1–5%, which is much better than the gel-based assays involving dinucleotide combinations [17]. Docking studies offered a molecular explanation in support of the efficient cleavage behavior for ApU, CpU bonds, reduced efficiency of UpU, and insignificant cleavage of GpU bonds (Figure 1 and Figure 3, Supplemental Figures S8 and S9). This is consistent with the alignment, orientation, and superimposition of the associated bases and ribose in the enzyme active site. Further, the docking poses also offer an explanation for the lack of cleavage upon chemical modification. For example, N1 methylation of adenosine restricted the cleavage of the [m^1^A]pU bond (Supplemental Figure S2). The N1-methyl group increases the size of the adenine by about 2.5 Å, which in turn interferes with the binding interactions between Thr43 and Ser44 and adenosine (Supplemental Figure S9). 

Uridine monophosphate (UMP), if formed upon cleavage of CpU and UpU in the -CpUpUpUpG- sequence, is not detectable by LC-MS. Its levels, however, could be low as the 5′ end digestion product—UACACCCGUAGGG*CU* (with CU instead of C at 3′ end), and 3′ end digestion products *UU*GAGA and *UUU*GAGA (with UU at 5′-end) were observed at high abundance (Figure 1). Molecular docking showed uridine turning to the right to form a hydrogen bond with Arg181 (Supplemental Figure S8), thereby pulling the 2′-OH of ribose forward. Further, the 5′ uracil in UpU does not form a hydrogen bond with Ser44 unlike cytosine in CpU, which could delay the positioning. These limitations are responsible for the reduced cleavage efficiency of the UpU bond.

A similar but more pronounced leftward shift and incorrect positioning of ribose phosphate and uridine in GpU as illustrated by the superimposition with CpU docking solution explain the insignificant cleavage for GpU (Supplemental Figure S9) or even Gp[D] or [m^7^G]pU sequences. These observations strongly suggest that the bonding contacts with the 5′ base of the dinucleotide also influence the RNA cleavage. In other words, interactions at the B1 and B2 sites determine the dinucleotide specificity of RNA cleavage by RNase MC1, as they can place or pull the phosphodiester bond from the catalytic site.

### 3.2. Cleavage Specificity and Efficiency of Cusativin

The semiquantitative LC-MS approach with a customized RNA sequence has documented the cleavage preferences of cusativin (CpA (70–90%), CpG (80–100%), CpU (80–100%), UpA (70–90%), UpU (14–30%), and ApU (14–30%) and no cleavage for the CpC bond) (Figure 2, Table 1). To help explain this behavior, we employed template-based modeling approaches to predict the cusativin tertiary structure and perform ligand docking. Such approaches have successfully revealed the molecular contacts with high accuracy in previous instances [21,22,23,24,39]. High sequence similarity with RNase MC1 (Supplemental Tables S2 and S3) enabled homology modeling of cusativin to predict the tertiary structure. Both protein models exhibited high structural similarity, saving specific loops and binding site residues (Supplemental Figure S10).

Cusativin-mediated cleavage of ApU bond can be explained by the stabilization of adenine (N7 and N3 of purine making H-bond with Ser70 and Arg176) and ribose phosphate in the B1 and catalytic sites. However, the orientation of adenine differs from cytosine in the B1 site (Supplemental Figure S15) as no H-bond can be seen for the amino group at the C6 position, unlike in RNase MC1 where Thr43 makes such a bond. Similarly, the alignment of the UpU docked pose with CpU showed near-exact overlap except for two positions. First, the uracil ring is flipped, leading to the opposite positioning of the carbonyl group (at C-2) to make an H-bond with Lys113. Secondly, 2′-OH of 5′ uridine pointed upward in all the docking poses (Supplemental Figure S16). Such positioning seems to result in lower cleavage of RNA at UpU and ApU bonds.

Higher levels of cleavage at UpA can be attributed to the strong interactions in the active site. Near identical positioning of uracil in B1 sites such as cytosine in CpU, strong H-bond interactions at N1 and N6 atoms of adenine with Asn97 and Gln36, coupled with another H-bond between the C6 amino group and Pro96, led to binding stabilization. Although this seems to be novel, proline is a conserved residue for the T2 family of proteins at this site [40]. Adenine also exhibited hydrophobic interactions with Phe106 and Val99, making it a perfect ligand for binding to the B2 site. Thus, the stable binding of UpA and optimal interactions with the catalytic site and low ∆G values were responsible for serving as an ideal substrate (Supplemental Figure S14). CpC, on the other hand, exhibited a low PLP score and a high ∆G value (for all docked poses analyzed), although it fits into the active site such as the CpU dimer. One critical difference could be the way the 3′ base binds to the B2 site. Although, Asn97 formed H-bond with C4 amino group of cytosine and dipole-dipole interaction between N3 of cytosine and amide oxygen, absence of strong hydrophobic interactions between cytosine and Val99 side chain (>4 Å distance) and lack of contacts with Gln36 and Val98 backbone could be responsible for lack of stable binding and cleavage (Supplemental Figure S12). Interestingly, Asn97Ser (N97S) mutation altered the cleavage characteristics of cusativin in ways comparable to the alteration of N71S [35] in MC1, suggesting similar roles in effecting cleavage specificity (Supplemental Figure S17). Similarly, the Thr100Arg (T100R) mutation also resulted in altered cleavage characteristics, suggesting a dynamic adjustment of both the B1 and B2 sites even though both mutations were in the B2 site. The observed cleavage behavior is also supported by additional H-bonding-based docking interactions of these dinucleotides in the mutant enzyme active site. However, the cleavage activity was reduced to a great extent in these mutants, as revealed by the steady-state enzyme kinetic analysis, indicating the adverse impact on the orientation of the cleavable phosphodiester bond in the catalytic site. Future experiments involving the characterization of the cusativin crystal structure can help shine a spotlight on these aspects.

### 3.3. Comparison of the Structure and Base Recognition by RNase MC1 and Cusativin

When a minimum of >5% cleavage is considered, MC1 cleaved RNA predominantly at ApU, CpU, UpU, and to some extent at CpA (10–20%) (Figure 1), whereas cusativin exhibited expanded substrate specificity with an overlap of cleavages (at CpU, CpG, CpA, UpA, UpU, and ApU) (Figure 2). A comparison of binding sites reveals that Thr43 in the B1 site of MC1 is replaced by arginine in cusativin (Table 2), causing its nonparticipation in binding. The loop from 178 to 181 (DCTR) of MC1 is substituted by the bigger loop (NCNKAR) in cusativin (Supplemental Figure S10), which in turn altered the orientation of Arg in cusativin (position 208), preventing the interaction. Furthermore, Lys178 of cusativin stabilizes the phosphate moiety at the 5′ end (B1), which is not the case for MC1. Replacement of glutamine in MC1 (at 164) by glutamate in cusativin (at 189) restricts the potential interactions in the B2 site. Substitution of leucine in MC1 (at 73) with valine in cusativin (at 99) in the B2 site reduces the side chain by 1.5 Å making room for purines to fit. Similarly, the replacement of Arg74 in MC1 with Thr in cusativin (at 100) also alters the side chain size and chemical nature. Although Arg74 does not take part in the binding of uracil by MC1, the side-chain hydroxyl group of Thr100 (cusativin numbering) donates a hydrogen bond to O6 of guanine. This may be one reason cusativin cleaves CpG and MC1 does not. Such an interpretation is supported by other MC1 mutants, N71T and N71S, which exhibit altered specificity for CpG cleavage instead of CpU [35].

## 4. Materials and Methods

### 4.1. RNA

A customized RNA (5′-GCAUCAGAAAUACACCCGUAGGGCUUU-GAGA-3′) with all the dinucleotide combinations (4^2^ = 16) and four homopolymer sequences was purchased from Integrated DNA Technology (IDT). Yeast tRNA^Phe^ was obtained from Sigma, and all other chemicals were procured from Thermo Fisher Scientific. RNase T1 was obtained from Worthington Biochemical Corporation. RNase MC1 [11] and cusativin [12] were purified on a nickel column using His-tag protein purification kit (EMD Millipore) following their expression from recombinant plasmids in Rosetta ^TM^ (DE3) and BL21 strains of *Escherichia coli,* as described before.

### 4.2. Cusativin Mutant Protein Preparation

Synthetic DNA (651 nt) sequences corresponding to N97S and T100R mutations of cusativin sequence were procured from IDT and cloned into the Bam HI and Hind III sites of protein expression plasmid, pET-22b. Recombinant clones were screened by colony PCR and the gene sequences confirmed by Sanger DNA sequencing at CCHMC (Cincinnati Children’s Hospital Medical Center) facility. Mutant protein expression and purification procedures were identical to the cusativin wild-type protein preparation [12].

### 4.3. RNA Digestion by Ribonucleases

About 50 units of RNase T1 were used to digest 1 µg of RNA at 37 °C for 2 h in the presence of ammonium acetate (120 mM, pH 6.5–7.0). In case of MC1 and cusativin, 0.25–1 µg enzyme was used to digest 1 µg of denatured RNA, with 120 mM ammonium acetate (pH not adjusted) at 37 °C and 60 °C for 2 h and dried in speedvac and stored at −20 °C until LC-MS injection.

### 4.4. LC-MS Analysis

The semi-quantitative liquid chromatography coupled with tandem mass spectrometry (LC-MS/MS) was performed through an ion-pairing reversed-phase liquid chromatography column connected to the Synapt G2-S mass spectrometer. The details of mobile phases, chromatography, and mass spectrometry conditions are described elsewhere [15,41]. The LC-MS/MS raw data containing sequence informative fragment ions of oligonucleotide digestion products were analyzed by RNA ModMapper [42,43]. Substrate specificity was computed by documenting the bond/s that should be cleaved to account for the observed oligonucleotide digestion products. Similarly, cleavage efficiency was scored based on the abundance (peak area) ratio of observed digestion product (*x*) and oligonucleotides that represent the undercut sequences (*y*) as percentage ($\;\frac{x}{x+y}\times 100)$. A minimum of 4 independent samples were analyzed to compute the range of mean values and standard deviation. Due to the semi-quantitative nature of the method, percentage cleavage efficiencies are categorized into four distinct levels (<5% cleavage is extremely low, 5–25% is low, 25–50% is moderate, and >50% as efficient cleavage).

### 4.5. Multiple Sequence Alignment

Ribonuclease sequences that exhibit highest similarities with cusativin were identified by NCBI BlastP (https://blast.ncbi.nlm.nih.gov/Blast.cgi?PAGE=Proteins) (accessed on 5 March 2020). Active site amino acid sequences of select T2 enzymes were aligned by MUSCLE (https://www.ebi.ac.uk/Tools/msa/muscle/) (accessed on 15 March 2020) tool [44].

### 4.6. Protein Modeling and Molecular Docking

A model tertiary structure for cusativin was prepared by homology modeling using SWISS-MODEL workspace (https://swissmodel.expasy.org/) (accessed on 5 May 2020) [45]. This exercise yielded RNase MC1 crystal structure, 1BK7 [34] as a top template suitable for generating the cusativin homology model. Ligands (dinucleotide and mononucleotides) were drawn using molview (https://molview.org/) (accessed on 17 August 2020) and saved as a 3D model. These molecules are then optimized for their energy using Avogadro software by choosing the feature, “Fixed atoms are movable” [46]. The prepared protein and ligands were subjected to molecular docking in slow mode to predict the binding affinity between protein and ligand using Genetic Optimization for Ligand Docking (Hermes GOLD (version 2020.2.0 Build 290188)) software developed by Cambridge crystallographic data center (CCDC) [47]. The default setting that allowed flexible ligand with different binding site conformations was used. The target protein’s site is chosen by selecting the protonated atom of catalytic histidine (H92 for RNase T1, H119 for RNase A, H34 for MC1, and H60 for cusativin) at the center of active site and all the atoms within 10 Å. The docking results were initially evaluated for binding of the ligand phosphate in the catalytic site and the 5′ base in the B1 site. Such poses were further scored for the number of hydrogen bonds made with active site residues, total free energy change upon ligand binding, and PLP score that predicts the binding affinity between the docked molecules [48].

The chemPLP scoring function is used to model the steric complementarity between protein and ligand. Further, the distance- and angle-dependent hydrogen and metal bonding terms from ChemScore (total free energy change upon ligand binding) are also considered [48] to estimate the internal torsion that occurs during the ligand-protein binding. The maximum number of conformations for the ligand was set to the default genetic algorithm (GA) settings, which generate 30,000 GA operations per five rotatable bonds of the ligand [48]. GOLD software docks each conformer of the ligand, and each pose of protein-ligand is ranked in terms of chemPLP fitness score. All the docking poses indicated by the GOLD were evaluated for proper configuration of ligand in the active site. Such poses were ranked based on high fitness scores and low ∆G values. All docking operations were performed at least five times, and each operation produced up to ten poses if they are 1.5 Å apart. Selected poses were imported to pymol (https://pymol.org) (accessed on 5 December 2020) for detailed analysis including the ligand alignment between the crystal structures and docked poses.

## 5. Conclusions

In this work, we detail the cleavage specificity and efficiency of RNase MC1 and cusativin by a semi-quantitative LC-MS analysis using a customized RNA. The sequence information of the observed digestion products reflects the accurate cleavage specificity and efficiency. The cleavage features are further explained by documenting the molecular interactions between the enzyme active site and dinucleotide ligands through homology modeling, docking studies, and targeted mutation studies. These studies support the dinucleotide-specific RNA cleavage nature of MC1 and cusativin. Such RNases serve as bioanalytical tools to locate post-transcriptional modifications in any RNA sequence, including mRNAs, through liquid chromatography coupled with tandem mass spectrometry (LC-MS/MS) analysis (or RNA modification mapping) [49]. Knowledge of the nucleobase-specific cleavage sites simplifies mass spectral analysis as it restricts the allowable base compositions for a specific mass measurement [50]; thus, playing an effective role in RNA modification mapping data analysis and interpretation [42,51].

## Figures and Tables

**Table 1:** RNA cleavage preferences of MC1 and cusativin. Cleaved dinucleotide bonds are shown.

NpN	% Cleavage	
	MC1	Cusativin
ApC	0–5	0
ApU	70–100	14–30
CpA	10–20	70–90
CpG	0–1	80–100
CpU	80–100	80–100
UpA	0–5	70–90
UpU	45–60	14–30
GpU	1–1.5	0
ApA	0–5	0

**Table 2:** Comparison of nucleobase binding site amino acid residues in RNase MC1 and cusativin. Substitutions in dark orange cells indicate significant changes in interactions, those in light orange cells do not show significant changes in interactions and those highlighted in yellow does not show interactions with the substrate.

RNase	Nucleotide Binding Site					
	B1 Site				B2 Site	
MC1	T43	Q164	R152	R181	L73	R74
Cusativin	R69	E189	K178	R208	V99	T100

## Data Availability

Required data files will be available upon request.

## Supplementary Materials

The following supporting information can be downloaded at: https://www.mdpi.com/article/10.3390/ijms23137021/s1.
