# Supplementary material for: RNA Cleavage Properties of Nucleobase-Specific RNase MC1 and Cusativin Are Determined by the Dinucleotide-Binding Interactions in the Enzyme-Active Site
Source: Int J Mol Sci. 2022 Jun 24;23(13):7021. doi: 10.3390/ijms23137021 (PMC9266746; doi:10.3390/ijms23137021)
Supplement: Supplementary file 1 [file ijms-23-07021-s001.zip › ijms-1765394-supplementary.pdf]

Supplemental information

RNA cleavage properties of nucleobase-specific RNase MC1 and cusativin are determined by the dinucleotide binding interactions in the enzyme active site

Priti Thakur, Jowad Atway, Patrick A. Limbach and Balasubrahmanyam Addepalli*

Rieveschl laboratories for mass spectrometry, Department of Chemistry, University of Cincinnati, Cincinnati OH 45221

* Corresponding author

Steady state enzyme kinetic analysis of cusativin, and its variants - T100R and N97S

Kinetic parameters were determined by monitoring the decrease in substrate RNA intensity when incubated with fixed amounts of purified protein preparations of cusativin. Increasing amounts of (0.5 to 4 μg) of yeast $tRNA^{Phe}$ was treated with 250 ng of wild type and mutant protein preparations at 60°C for 80 minutes. No enzyme samples were also treated under same conditions while keeping the volume constant with buffer. Following incubation, samples were diluted with RNA sample loading buffer (Sigma) and heated at 65 °C for min, chilled on ice, and added 1 μL of diluted (1:25) SYBR™ Gold stain before subjecting to 15% polyacrylamide-8M urea gel electrophoresis (Urea-PAGE) in 0.5X TBE buffer. The gels were photographed on a UV transilluminator, and the intensity of substrate RNA band is quantified using the ImageJ software. The product is distinguished from a substrate by inspecting the intensity of the main RNA band. Upon cleavage of RNA by the enzyme, the main RNA band will have lower intensity than the band corresponding to the same amount of yeast $tRNA^{Phe}$ that is not treated. Amount of product formed was calculated by subtracting the band intensity of substrate RNA in the digested samples from respective undigested samples. K_m and V_{max} value were determined via Lineweaver-Burk plots, and a comparison of the K_m and K_m/K_{cat} values of mutants is made.

Table S1 – Comparison of the docking interactions between the mononucleotides (3'-GMP or 5'-GMP) or dinucleotide ligands and the active site amino acid residues of RNase T1 with those observed in crystal structures, 1RGA and 1B2M.

Rnase T1			# H-Bonds			Hydrophobic interactions	ΔG kcal/mol
**Ligand**	**PLP_Score**	**RMSD**	**5' Base**	**Ribose phosphate**	**3' Base**		
1RGA_3GMP	-	-	7	8	-	2	
3'-GMP	72	0.18	7	8	-	2	-20.1
5'-GMP	71	0.19	7	5	-	2	-19.2
1B2M_GpU	-	-	7	4	0	2	-5.8
GpU	99.4	0.26	7	7	2	2	-18.1
ApU	80.08	0.18	2	6	2	1	-7.3
CpU	60.6	0.63	2	6	1	1	-5.40

Table S2 – Comparison of the docking interactions between the dinucleotide ligands and the active site amino acid residues of RNase A with those observed in crystal structure, 1RPG.

RNase A			# H-Bonds			Hydrophobic interactions	ΔG kcal/mol
**Ligand**	**PLP Score**	**RMSD**	**5' Base**	**Ribose phosphate**	**3'Base**		
1RPG_CpA			2	7	2	1	-
CpA	74.8	0.26	5	5	2	1	-16.2
UpA	74.2	0.18	2	7	3	1	-8.1
GpA	59.5	0.18	2	3	1		1.5

Table S3: Docking **i**nteractions of the active site amino acid residues of MC1 with dinucleotide ligands.

Interactions between the dinucleotide ligands and the active site amino acid residues of MC1 were scored following molecular docking with CCDC GOLD software**.** y denotes positive interaction and n denotes no interaction)

		B1 site										B2 site						Catalytic site					
		Base and ribose							phosphate			Base and ribose						proton donor/ acceptor		Transition state stabilization			
Ligand	PLP	S44	R150	R181	T43	G42	R150	K87	R150	W37	Q164	Q9	N71	V72	L73	R74	F80	H34	H88	E84	K87	H83	ΔG
1UCD_5U MP	NA	NA					Sug=O	2'OH	o –Ph	π-π	o -	O4 H-bond	N3 H-bond	O4	Base Hydrophobic	O2 H-bond	π-π	PO-CH25'	P=O	Not bonded	PO^-	PO^-	
CpU	73.9	y	y	y	-		y	y	y	Y	y	y	y	y	y	n	y	n	n	y	y	y	-12.3
UpU	69.39	n	y	y	-		n	y	y	Y	n	y	y	y	y	n	y	y	y	n	y	y	-8.98
ApU	69.36	y	n	n	y	-	y	y	y	Y	y	n	y	y	y	n	y	n	y	n	y	y	-5.95
GpU	55.45	y	n	y	y	y	y	y	y	y	y	y	n	n	y	n	y	y	n	n	y	n	-1.65

Table S4: Comparison of the docking interaction scores of dinucleotide ligands with the active site amino acid residues of RNase MC1.

MC1			Number of H-Bonds				
Ligand	PLP_Score	RMSD	5' Base	Ribose phosphate	3'Base	Hydrophobic interactions	ΔG
**CpU**	73.9	0.31	4	9	4	2	-12.3
**UpU**	69.39	0.26	3	6	4	2	-8.98
**ApU**	69.36	0.14	2	7	4	2	-5.95
**GpU**	55.45	0.31	5	3	1	1	-1.65

Table S5 : BLASTP search analysis of cusativin amino acid sequence.

Description	Scientific Name	Max Score	Total Score	Query Cover	E value	Percentage identity	Acc. Len	Accession (PDB ID)
RIBONUCLEASE MC1 FROM THE SEEDS OF BITTER GOURD [*Momordica charantia*]	*Momordica charantia*	224	224	87%	2.00E-74	**53.12**	190	**1BK7_A**
Crystal structure of the RNase MC1 mutant N71T in complex with 5'-GMP [*Momordica charantia*]	*Momordica charantia*	223	223	87%	3.00E-74	**53.12**	191	1J1F_A
Crystal structure of the RNase MC1 mutant Y101A in complex with 5'-UMP [*Momordica charantia*]	*Momordica charantia*	223	223	87%	8.00E-74	**53.12**	197	1V9H_A
Crystal structure of the RNase MC1 mutant N71S in complex with 5'-GMP [*Momordica charantia*]	*Momordica charantia*	222	222	87%	1.00E-73	52.6	190	1J1G_A
Crystal structure of Ribonuclease MC1 N71T mutant [*Momordica charantia*]	*Momordica charantia*	220	220	87%	6.00E-73	52.08	190	1UCG_A
Crystal Structure of the Nicotiana glutinosa Ribonuclease NW [*Nicotiana glutinosa*]	*Nicotiana glutinosa*	134	134	88%	7.00E-39	34.83	208	1IYB_A
Crystal Structure Of Rnase Le [*Solanum lycopersicum*]	*Solanum lycopersicum*	127	127	82%	3.00E-36	35.64	208	1DIX_A
Crystal structure of the RNase NT in complex with 5'-GMP [*Nicotiana glutinosa*]	*Nicotiana glutinosa*	109	109	82%	3.00E-29	31.91	217	1VCZ_A
Crystal Structure of Japanese pear S3-RNase [*Pyrus pyrifolia*]	*Pyrus pyrifolia*	86.7	86.7	81%	1.00E-20	30.77	200	1IQQ_A
Crystal Structure Of Nicotiana Alata Gemetophytic Self-incompatibility Associated Sf11-rnase [*Nicotiana alata*]	*Nicotiana alata*	81.6	81.6	82%	8.00E-19	25.95	196	1IOO_A
Crystal Structure Analysis of Human RNase T2 [*Homo sapiens*]	*Homo sapiens*	82	82	80%	1.00E-18	28.96	238	3T0O_A
THE CRYSTAL STRUCTURE OF RIBONUCLEASE RH FROM RHIZOPUS NIVEUS AT 2.0 A RESOLUTION [*Rhizopus niveus*]	*Rhizopus niveus*	75.1	75.1	61%	4.00E-16	29.87	222	1BOL_A
The three-dimensional structure and X-ray sequence reveal that trichomaglin is a novel S-like ribonuclease [*Trichosanthes lepiniana*]	*Trichosanthes lepiniana*	70.9	70.9	79%	1.00E-14	28.04	209	1SGL_A
The 1.7A crystal structure of Actibind a T2 ribonucleases as antitumorigenic agents [*Aspergillus niger*]	*Aspergillus niger*	56.6	56.6	61%	3.00E-09	24.39	237	3TBJ_A
Crystal structure of Actibind a T2 RNase [*Aspergillus niger*]	*Aspergillus niger*	56.2	56.2	61%	3.00E-09	24.39	247	3D3Z_A

Table S6 : Template RNase proteins for modeling cusativin.

Homology modeling templets (18) generated by the SWISS-MODEL for Cusativin modeling						
RNases	Protein description	Identity	Method	Oligo State	Ligands	Template
MC1	Crystal structure of the RNase MC1 mutant N71T in complex with 5'-GMP	52.66	X-ray, 1.6Å	monomer	1 x 5GP	1j1f.1.A
MC1	Crystal structure of the RNase MC1 mutant Y101A in complex with 5'-UMP	52.66	X-ray, 2.0Å	monomer	1 x U5P	1v9h.1.A
MC1	Crystal structure of the RNase MC1 mutant N71T in complex with 5'-GMP	54.26	X-ray, 1.6Å	monomer	1 x 5GP	1j1f.1.A
MC1	Crystal structure of Ribonuclease MC1 from bitter gourd seeds complexed with 5'-UMP	52.66	X-ray, 1.3Å	monomer	1 x U5P, 1 x URA	1ucd.1.A
MC1	Crystal structure of the RNase MC1 mutant Y101A in complex with 5'-UMP	54.26	X-ray, 2.0Å	monomer	1 x U5P	1v9h.1.A
MC1	Crystal structure of Ribonuclease MC1 N71T mutant	53.19	X-ray, 1.6Å	monomer	2 x MN	1ucg.1.A
MC1	Crystal structure of Ribonuclease MC1 from bitter gourd seeds complexed with 5'-UMP	54.26	X-ray, 1.3Å	monomer	1 x U5P, 1 x URA	1ucd.1.A
MC1	Crystal structure of the RNase MC1 mutant N71S in complex with 5'-GMP	54.01	X-ray, 1.6Å	monomer	2 x 5GP	1j1g.1.A
NW	Crystal Structure of the Nicotiana glutinosa Ribonuclease NW	35.75	X-ray, 1.5Å	homo-dimer	2 x 5GP	1iyb.1.B
NW	Crystal Structure of the Nicotiana glutinosa Ribonuclease NW	35.75	X-ray, 1.5Å	homo-dimer	2 x 5GP	1iyb.1.A
NW	Crystal Structure of the Nicotiana glutinosa Ribonuclease NW	36.27	X-ray, 1.5Å	homo-dimer	2 x 5GP	1iyb.1.A
NW	Crystal Structure of the Nicotiana glutinosa Ribonuclease NW	36.27	X-ray, 1.5Å	homo-dimer	2 x 5GP	1iyb.1.B
LE	CRYSTAL STRUCTURE OF RNASE LE	35.42	X-ray, 1.6Å	monomer	None	1dix.1.A
LE	CRYSTAL STRUCTURE OF RNASE LE	36.87	X-ray, 1.6Å	monomer	None	1dix.1.A
NT	Crystal structure of the RNase NT in complex with 5'-GMP	33.71	X-ray, 1.8Å	monomer	2 x 5GP	1vcz.1.A
RH	THE CRYSTAL STRUCTURE OF RIBONUCLEASE RH FROM RHIZOPUS NIVEUS AT 2.0 A RESOLUTION	35.11	X-ray, 2.0Å	monomer	None	1bol.1.A
T2	The 1.7A crystal structure of Actibind a T2 ribonucleases as antitumorigenic agents	31.5	X-ray, 1.8Å	monomer	1 x NAG-NAG, 1 x NAG	3tbj.1.A
Actibind	Crystal structure of Actibind a T2 RNase	31.5	X-ray, 1.7Å	monomer	1 x NAG-NAG, 1 x NAG, 1 x D3Z	3d3z.1.A

Table S7: Interactions of amino acid residues in the cusativin active site with dinucleotide ligands. Interactions between the dinucleotide ligands and the active site amino acid residues of cusativin were scored following molecular docking with CCDC GOLD software**.** Y denotes positive interactions; n denotes no interactions.

		B1 site (5')										B2 site (3')							catalytic site					
		Base and ribose							ribose PO_4			Base and ribose							proton donor/ acceptor	Transition state stabilization				
Ligand	PLP	R176	W 63	Q65	S70	K113	R176	E189	R176	E189	K178	Q36	N97 N3	N97 C4	V98	V99	T100	F106	H60	H109	E110	K113	H114	ΔG
CpU	78.4	y	y	n	y	y	y	y	y	y	y	y	y	y	y	y	n	y	y	y	y	y	y	-11.43
CpA	50.1	y	y	n	y	y	y	y	y	y	y	y	Y	n	n	y	n	y	y	y	y	y	y	-2.5
CpG	57.2	y	y	n	y	y	y	n	y	y	y	w	y	y	n	y	y	y	y	w	y	y	w	-6.16
CpC	46.6	y	n	n	y	y	y	n	y	y	y	w	n	y	y	w	n	y	n	n	n	y	n	-1.97
UpA	65	y	y	n	y	y	y	n	y	y	y	y	y	n	w	y	n	y	y	y	n	y	y	-7.69
UpU	54.4	n	n	y	n	y	y	n	y	n	y	n	y	y	y	w	n	y	y	n	n	n	n	-2.86
ApU	70.2	y	n	n	y	y	y	n	y	y	y	n	y	y	n	y	n	y	y	n	n	n	n	-4.45

Table S8: Comparison of the docking interaction scores of dinucleotide ligands with the active site amino acid residues of cusativin

Cusativin			Number of H-Bonds				
Ligand	PLP_Score	RMSD	5' Base	Ph	3'Base	π-π	ΔG
**CpU**	78.4	0.24	3	9	4	2	**-11.43**
**CpA**	50.1	0.22	2	8	2	2	**-2.5**
**CpG**	57.2	0.36	2	9	3	2	**-6.16**
**CpC**	46.6	0.31	2	6	2	1	**-1.97**
**UpA**	65	0.17	2	8	3	2	**-7.69**
**UpU**	54.4	0.23	2	3	4	1	**-2.86**
**ApU**	70.2	0.23	2	4	3	2	**-4.45**

Table S9: Enzyme kinetic analysis of cusativin and its genetic variants T100R and N97S

**Kinetic parameter**	**WT**	**T100R mt**	**N97S**
Km (μM)	21.8	34.7	18.6
Vmax (μM min^{-1})	0.32	0.47	0.19
Kcat (min^{-1})	0.74	1.08	0.43
Kcat/Km (M^{-1} min^{-1})	34.2	31.3	25.5

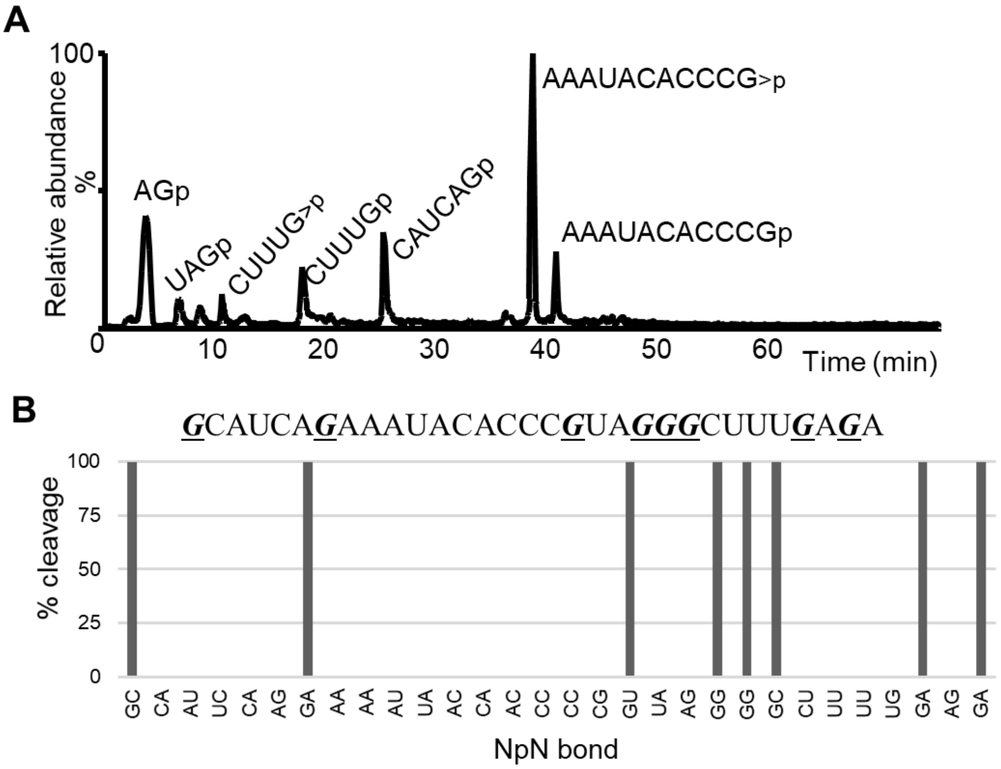

Figure S1. LC-MS based detection of RNase T1 digestion products from custom-made 31-nt RNA.

A. Total ion chromatogram (TIC) annotated with the observed digestion products containing linear (p) or cyclic (>p) phosphates. **B.** Positions of RNA cleavage in the sequence is underlined and shown in bold and italicized font. Cleavage efficiency was calculated by computing the percentage of the ratio between the peak area of expected digestion product and undercut oligonucleotide sequences.

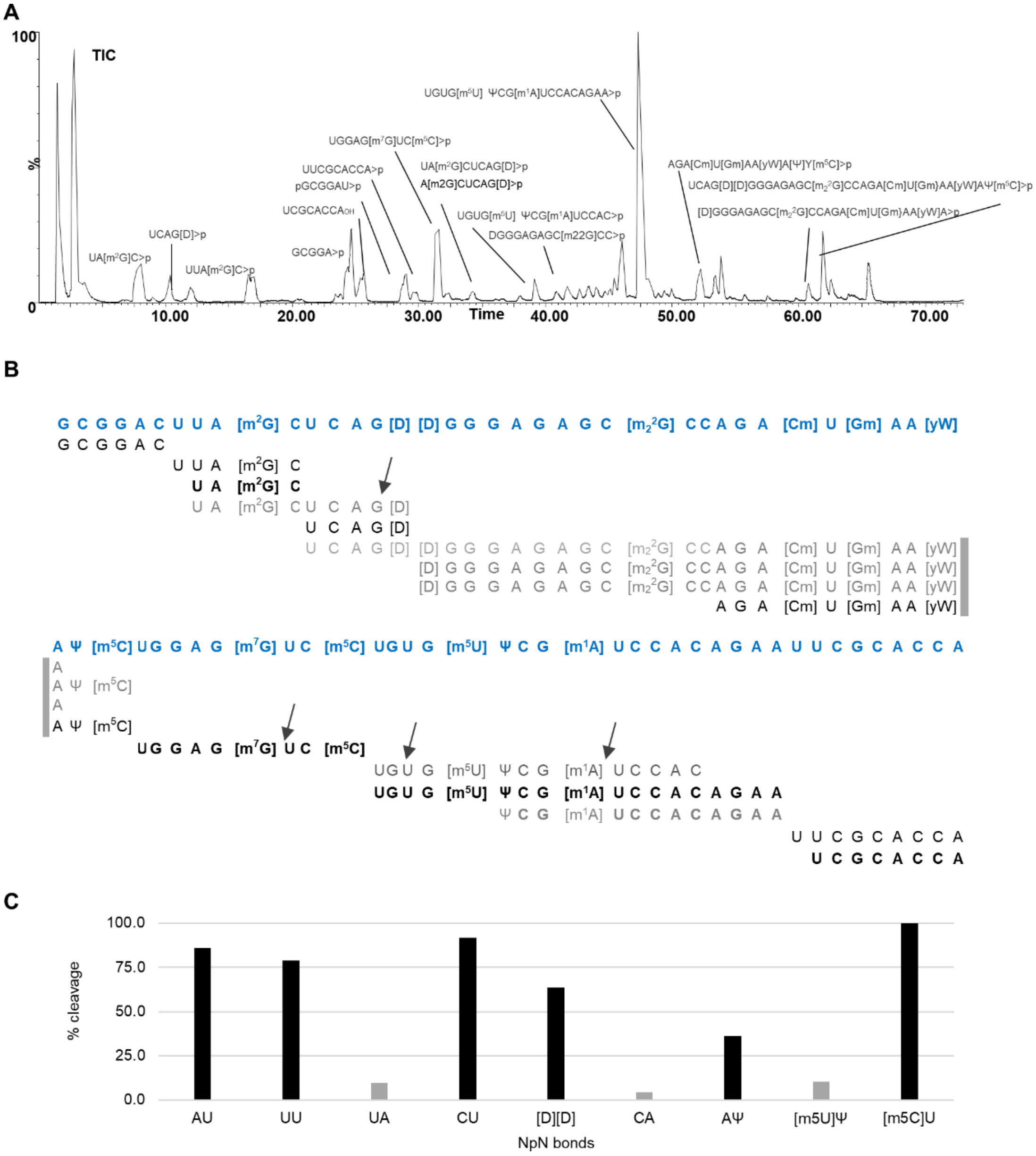

Figure S2. RNase MC1 digestion products of yeast tRNA^{Phe}.

A. TIC of the observed digestion products annotated for their sequences. **B.** Overlay of the observed digestion products of yeast tRNA^{Phe} mapped to the sequence. Arrows indicate the locations where cleavage was absent when uridine or dihydrouridine [D] was preceded by guanosine or its modification. Absence of overlaps for a given cleavage point (for example, [m^{5}C][U]) indicate 100% cleavage. **C.** Dinucleotide bonds of yeast tRNA^{Phe} cleaved by the RNase MC1. Bars represent the cleavage percentage of NpU (black) and NpN (gray) bonds

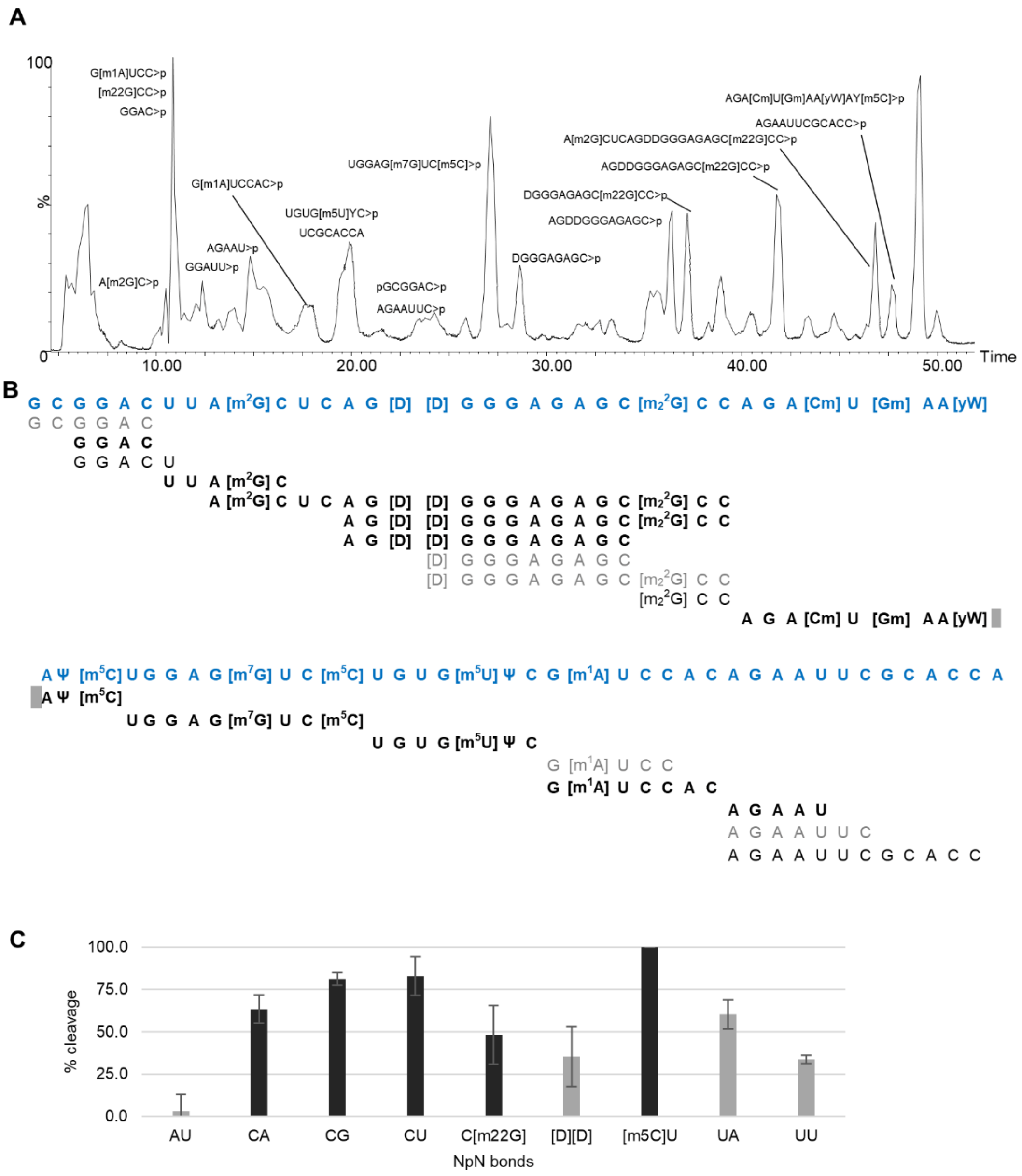

Figure S3. RNase cusativin digestion products of yeast tRNA^{Phe}.

A. TIC of the observed digestion products annotated for their sequences. **B.** Overlay of the observed digestion products of yeast tRNA^{Phe} were mapped to the sequence. Absence of overlaps for a given cleavage point (for example, [m^{5}C][U]) indicate 100% cleavage. **C**, Dinucleotide bonds of yeast tRNA^{Phe} cleaved by the RNase cusativin. Bars represent the cleavage percentage of CpN (black) and NpN (gray) bonds.

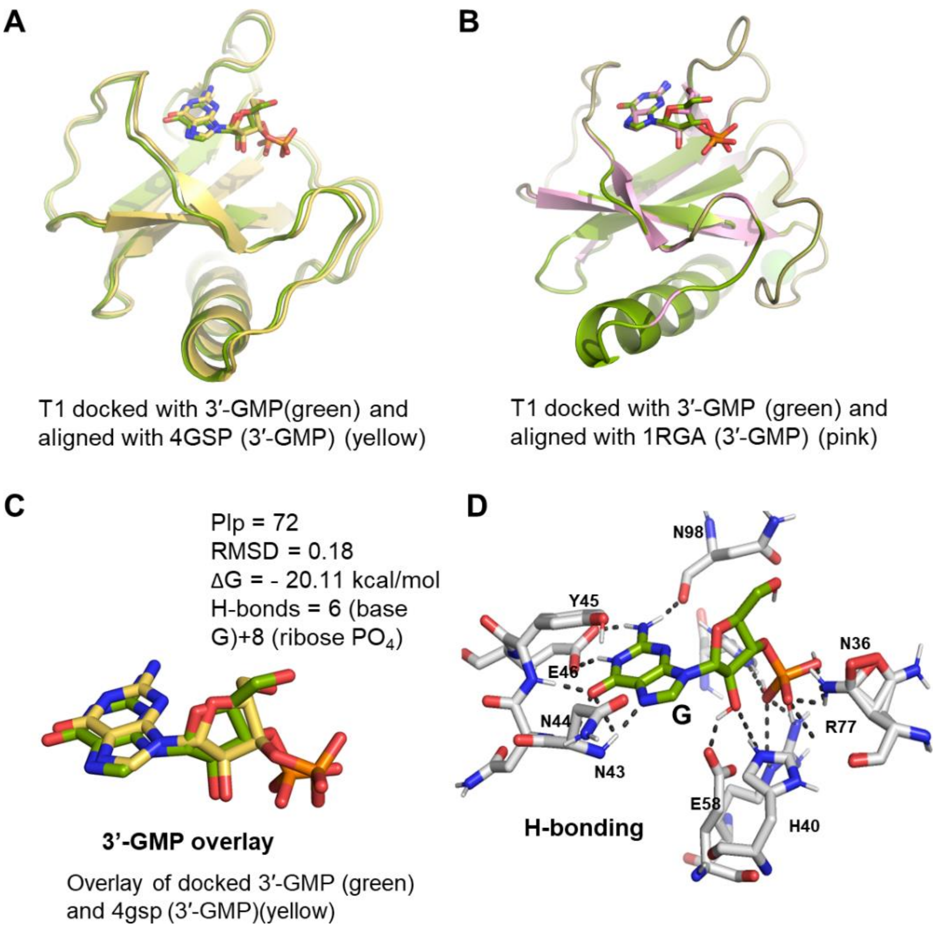

Figure S4. Overlay of the homology molecular model of RNase T1 docked with 3′-GMP and T1 crystal structure bound with 3′-GMP.

A. & B. Overlay of the RNase T1 docked with 3′-GMP (sea green) against the crystal structures of RNase T1 bound with 3′-GMP (yellow) (PDB ID: 4GSP), and (PDB ID: 1RGA). **C.** Overlay of the docked 3′-GMP (green) against the 3′-GMP found in the crystal structure (yellow)(PDB ID: 4GSP) is shown. **D.** Hydrogen bonding arrangement of the docked 3′-GMP (green) with the amino acid residues of the active site.

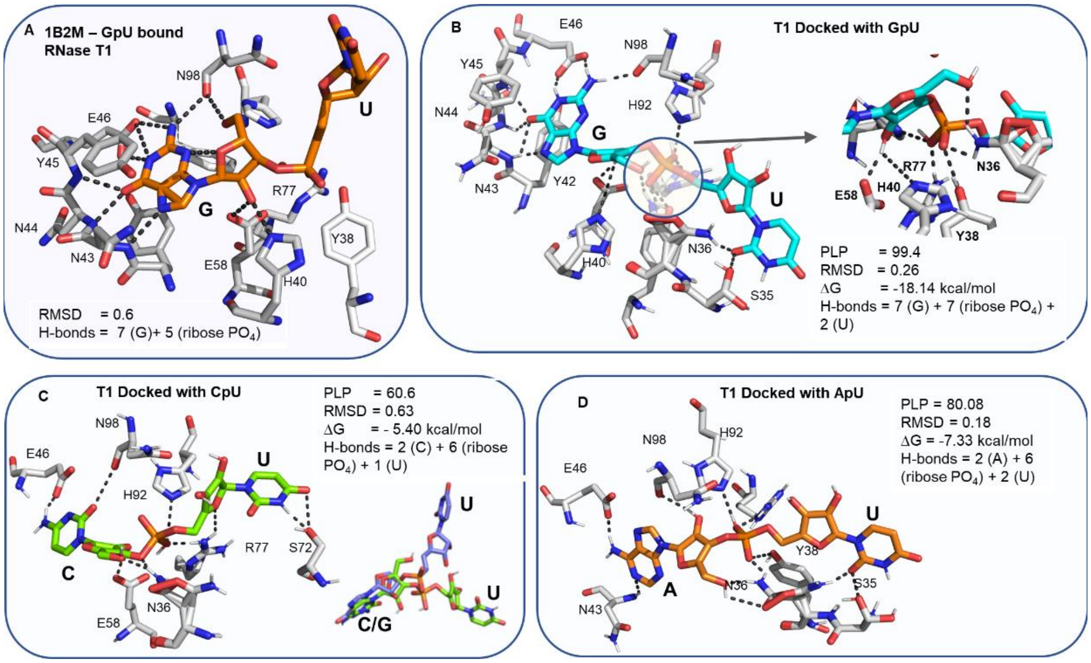

Figure S5. Molecular docking of the RNase T1 homology model with dinucleotide ligands.

A. Close-up view of the RNase T1 active site bound with GpU in the crystal structure (PDB ID 1B2M). **B.** RNase T1 homology model docked with GpU exhibited high fitness score. Zoomed view of catalytic region showing all the expected interactions. **C.** RNase T1 docked with CpU ligand (green) exhibited decreased fitness score. Docked ligand was also aligned with the T1 crystalized with GpU (purple) (PDB ID 1B2M) to illustrate the altered orientation for lack of cleavage. **D.** RNase T1 docked with ApU exhibited lower fitness score (PLP: 80, ΔG: -7.3 and fewer H-bonds with the nucleobases.

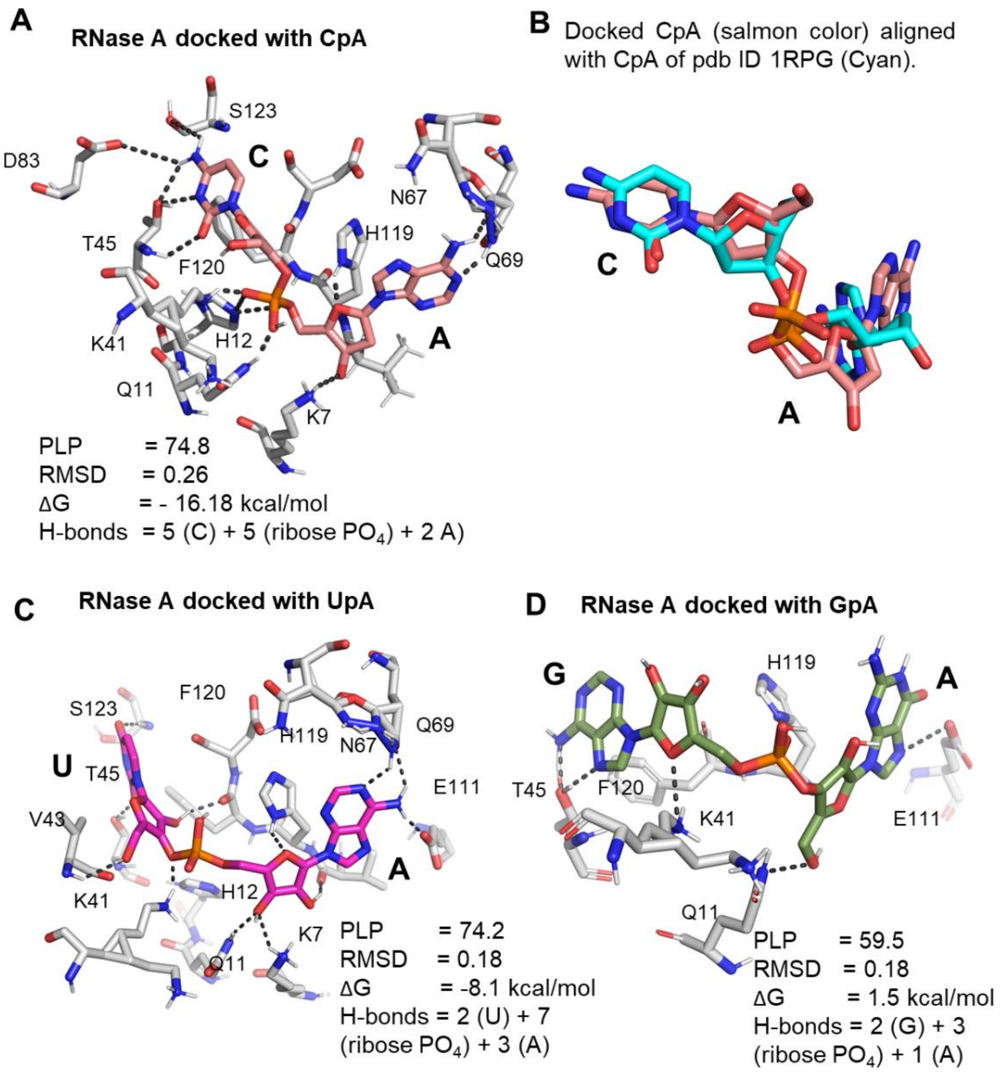

Figure S6. Molecular docking of RNase A with dinucleotide ligands.

A. RNase A homology model docked with CpA (salmon color) exhibited high fitness score. **B.** Overlay of the CpA (salmon) docked to RNase A with the deoxy(CpA) (cyan) bound to the RNase A active site in the crystal structure (PDB ID: 1RPG) **C.** RNase A docked with UpA ligand (pink). **D.** RNase A docked with GpA (green), where A bound to B1 site and G bound to B2 site, also exhibited lower fitness score.

A

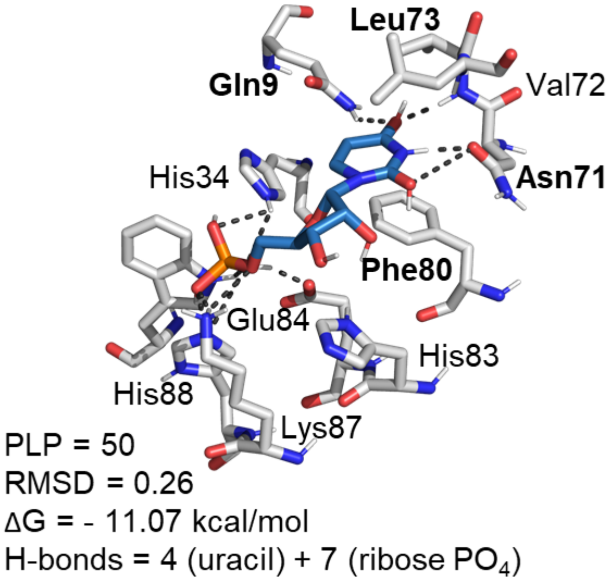

B

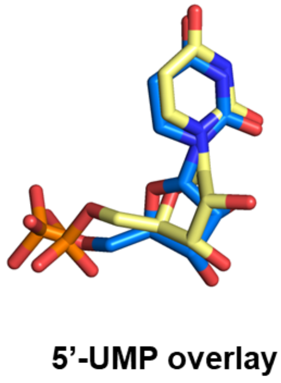

C

Amino acid	Bonds formed	1UCD Crystal	MC1_5UMP Docked
Gln9	O4 H-bond	3.0	3.0
Asn71	N3 H-bond	2.8	2.9
Leu73	π-π	3.6-3.7	3.6-3.7
Phe80	π-π	3.9	3.7-4.5
Val72	O4	2.9	2.9
Arg74	O2 H-bond	3.3	3.1

Figure S7. Molecular docking of RNase MC1 homology model with 5'-UMP ligand.

A. RNase MC1 docked with 5'-UMP (blue) exhibited high fitness score. **B**. The 5'-UMP ligand bound to the MC1 in the crystal structure, 1BK7, aligned well with the docked ligand. **C.** Near identical interactions observed in both crystal structure and docked ligand are indicated in the table format.

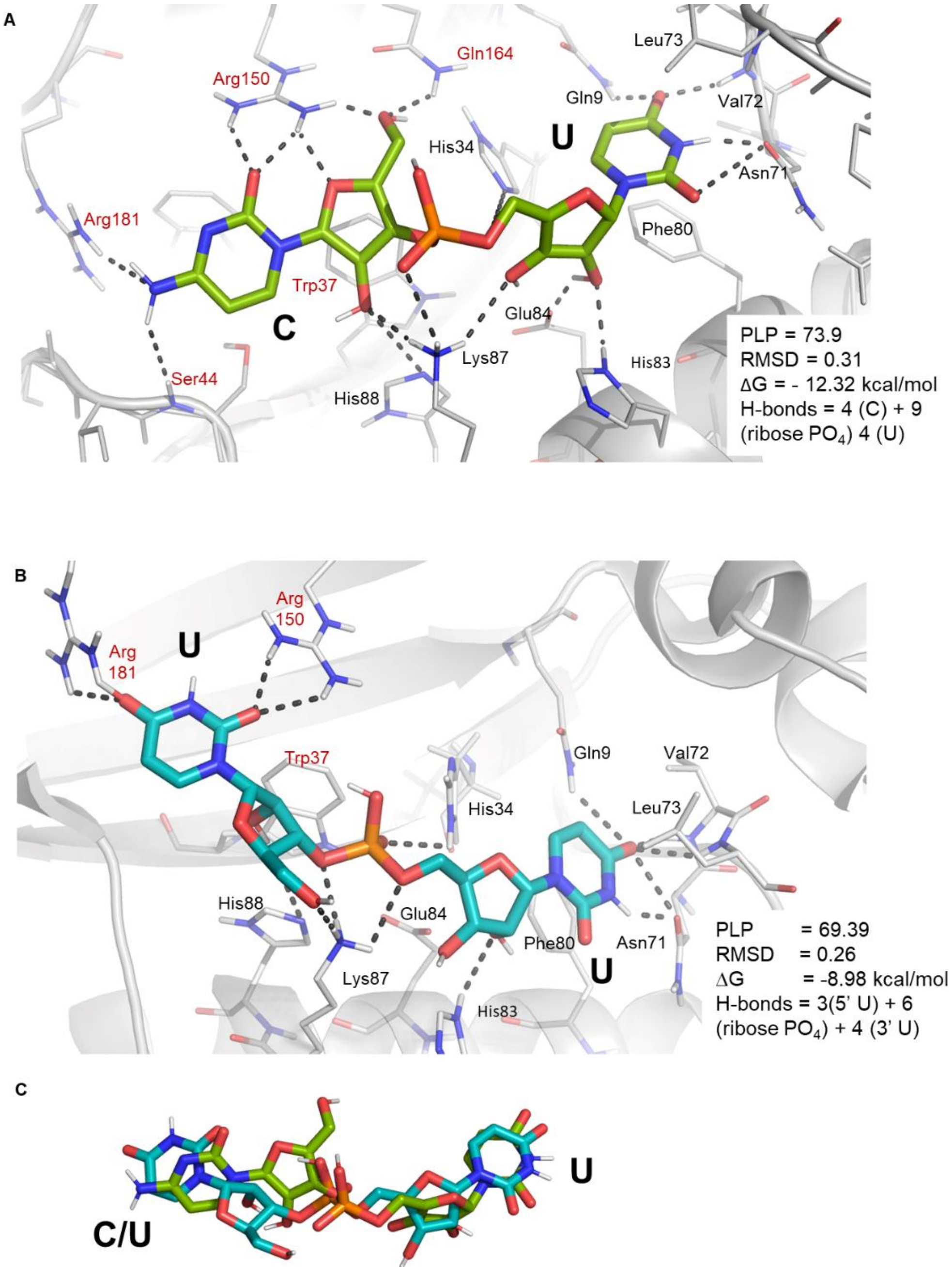

Figure S8. Molecular docking of RNase MC1 with CpU and UpU dinucleotide ligands.

RNase MC1 docked with CpU (**A**) and UpU (**B**) dinucleotides. Amino acid residues indicated in red represent interactions in the B1 site and are being reported first time. **C.** Alignment of the docked ligands CpU (green) and UpU (cyan) from the docked solutions.

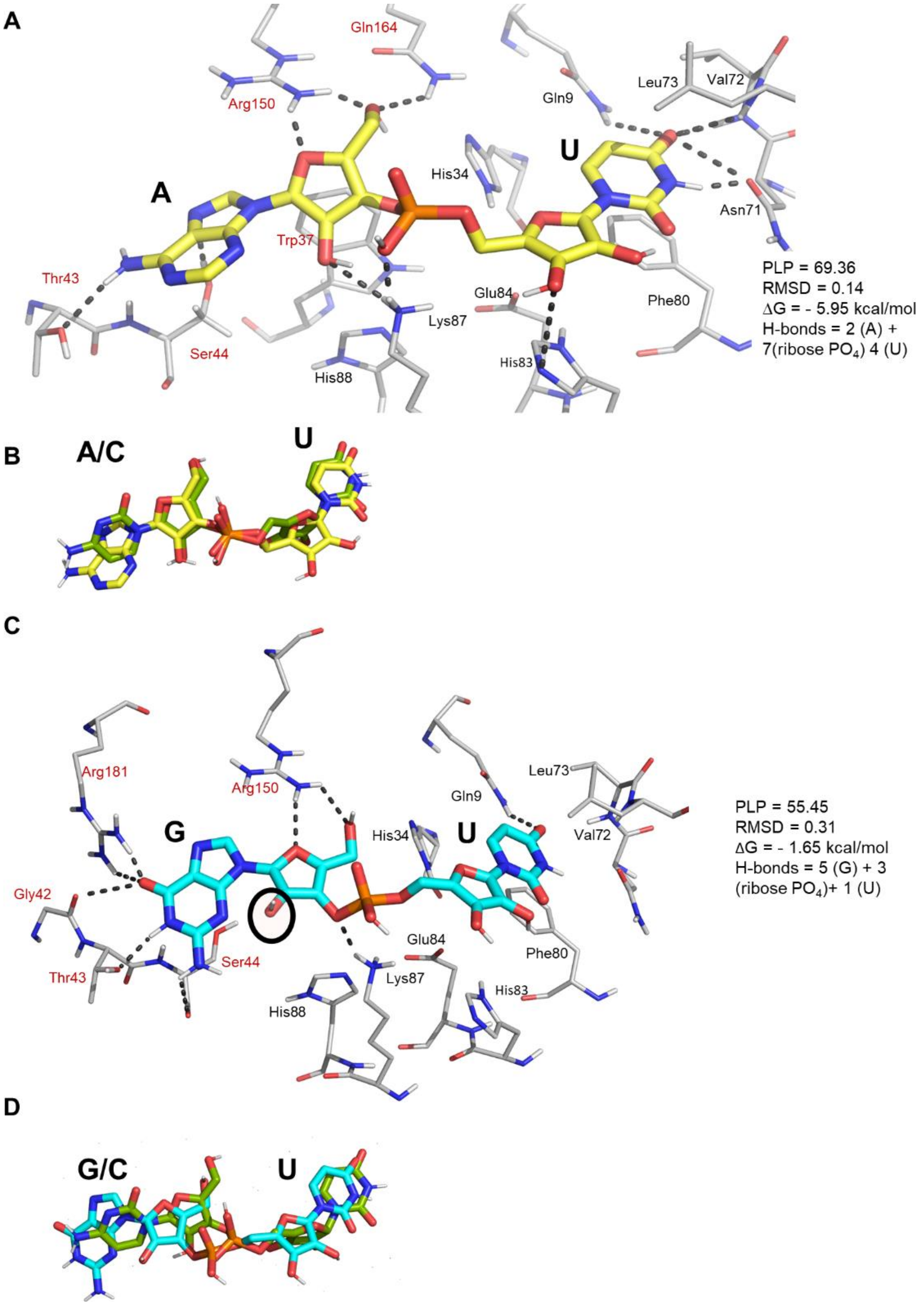

Figure S9. Molecular docking of RNase MC1 with ApU and GpU dinucleotide ligands.

A. RNase MC1 docked with ApU. The complex shows strong hydrogen bond interactions with active site residues. **B**. Structural alignment of the ApU (yellow) and CpU (green) ligands. **C** RNase MC1 docked with GpU dinucleotide, The complex exhibits fewer H-bonding interactions Black circle highlights the absence of bonding interactions involving the 2’-OH of ribose moiety. **D**. Overlay of the docked solutions of GpU (Cyan) and CpU (green) . Residues depicted in red belong to the binding site B1 of RNase MC1

A

```
MC1                ----------------------------FDSFWFVQQWPPAVCSFQKSGSCPGSGLRTFTI 33
Cusativin       1 MEKTKSVDVVFFVFVLTILFPIVKSQTFDDFWFVQQWPPAVCTLQ-SGRCVGRGTRSFTI 59
                                              **.************::* ** * * * *:***

MC1            34 HGLWPQQSGTSLTNCPGSPFDITKISHLQSQLNTLWPNVLRANNQQFWSHEWTKHGTCSE 93
Cusativin      60 HGLWPQKGGRSVTNCTGNQFDFTKIAHLENDLNVVWPNVVTGNNKFFWGHEWNKHGICSE 119
                  ******:.* *:***.*. **:***:**:.:**.:****: .**: **.***.*** ***

MC1            94 STFNQAAYFKLAVDMRNNYDIIGALRPHAAGPNGRTKSRQAIKGFLKAKFGKFPGLRCRT 153
Cusativin     120 SKFDEAKYFQTAINMRHGIDLLSVLRTGGVGPNGASKAKQRVETAISSHFGKDPILRCKK 179
                  *.*::* **: *::**:. *::..**. ..**** :*:.* ::  :.::*** * ***..

MC1           154 DPQTKVSYLVQVVACFAQDGSTLIDC--TRDTCGANFIF 191
Cusativin     180 ASNGQV-LLTEIVMCFDDDGVTLINCNKARSNCAGSFIF 217
```

B

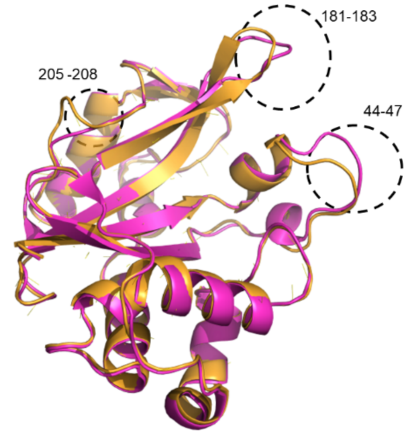

C

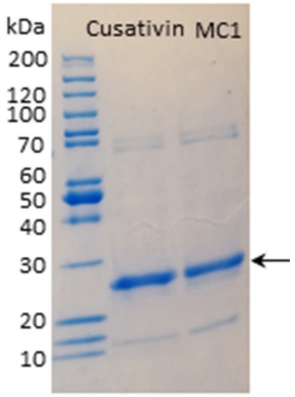

D

Sequence alignment of the active site residues of RNase cusativin, MC1, LE, NW and Rh

```
              36                   65    70            97    100                                          Gen Bank ID
Cus:   FWFVQQWP****  TIHGLWPQK-GGRSVTN--**  WPNVV--TGNNKFFWGHEWNKHGIC***LRCK**VL-LTEI***  AKU36755.1
MC1:   FWFVQQWP****  TIHGLWPQQ-SGTSLTN--**  WPNVL--RANNQQFWSHEWTKHGTC***LRCR**VSYLVQV***  AAB32233.1
LE:    FYFVQQWP****  GIHGLWPNNNDGTYPSN--**  WPTLACPSGSGSTFWSHEWEKHGTC***IQCN**SQ-LYQV***  MW843818.1
NW:    FYFVQQWP****  GIHGLWPNNNDGSYPSN--**  WPTLACPSGTGSAFWSHEWEKHGTC***IECN**SQ-LYQI***  BAC77613.1
Rh:    VVLNMQWA****  TLHGLWPDKCSGAYAPSGG**  WPSN---QGNNNVFWSHEWSKHGTC***IDCS**VA----L***  D00238.1
       . :   **. .    :*****:: .*    ..    **.     ....   *.*** ***       : *
          B2             B1     B1            B2         B2     P site
```

Figure S10. Homology modeling of cusativin and multiple sequence alignment of its active site with that of T2 family proteins. **A**. Sequence alignment of cusativin with MC1 depicting identities (102/192(53%)), positives (134/192 (69%)), gaps (4/192(2%)). **B**. Superimposition of cusativin homology model (yellow) with the MC1 crystal structure (PDB ID 1BK7 (pink) (RMSD: 0.37). The 3D-structure of cusativin was modeled by homology modeling and aligned with 1BK7 of MC1 in the pymol. Circles represent the mismatched regions. They include loops comprising 44-47, 181-183, and 205-208 residues indicating conformational differences for cusativin compared to RNase MC1. **C**. Sodium dodecyl sulfate-

polyacrylamide gel electrophoresis (SDS-PAGE) of cusativin and MC1. Following purification on Ni^{+}-agarose beads, ~3 µg of the protein preparation was electrophoresed through a 4-20% polyacrylamide gradient gel after denaturation with Laemmli buffer and stained with Coomassie brilliant blue. The size standards ranging from 10-200 kDa was indicated. Expected polypeptide with an approximate molecular weight of ~26 kDa is indicated by an arrow for both proteins **D**. Multiple sequence alignment of the active site regions of RNases Cusativin (*Cucumis sativus*, AAB32233.1), MC1(AKU36755.1), LE (*Lycopersicon esculentum,* MW843818.1), NW (from *Nicotiana glutinosa,* BAC77613.1), and Rh (*Rhizopus niveus* D00238.1) were aligned by the MUSCLE alignment tool[1]: Yellow highlighted residues are conserved in members of the RNase T2 family. Green, blue and gray highlighted residues represent the amino acid residues involved in base recognition. P-site refers to the catalytic site

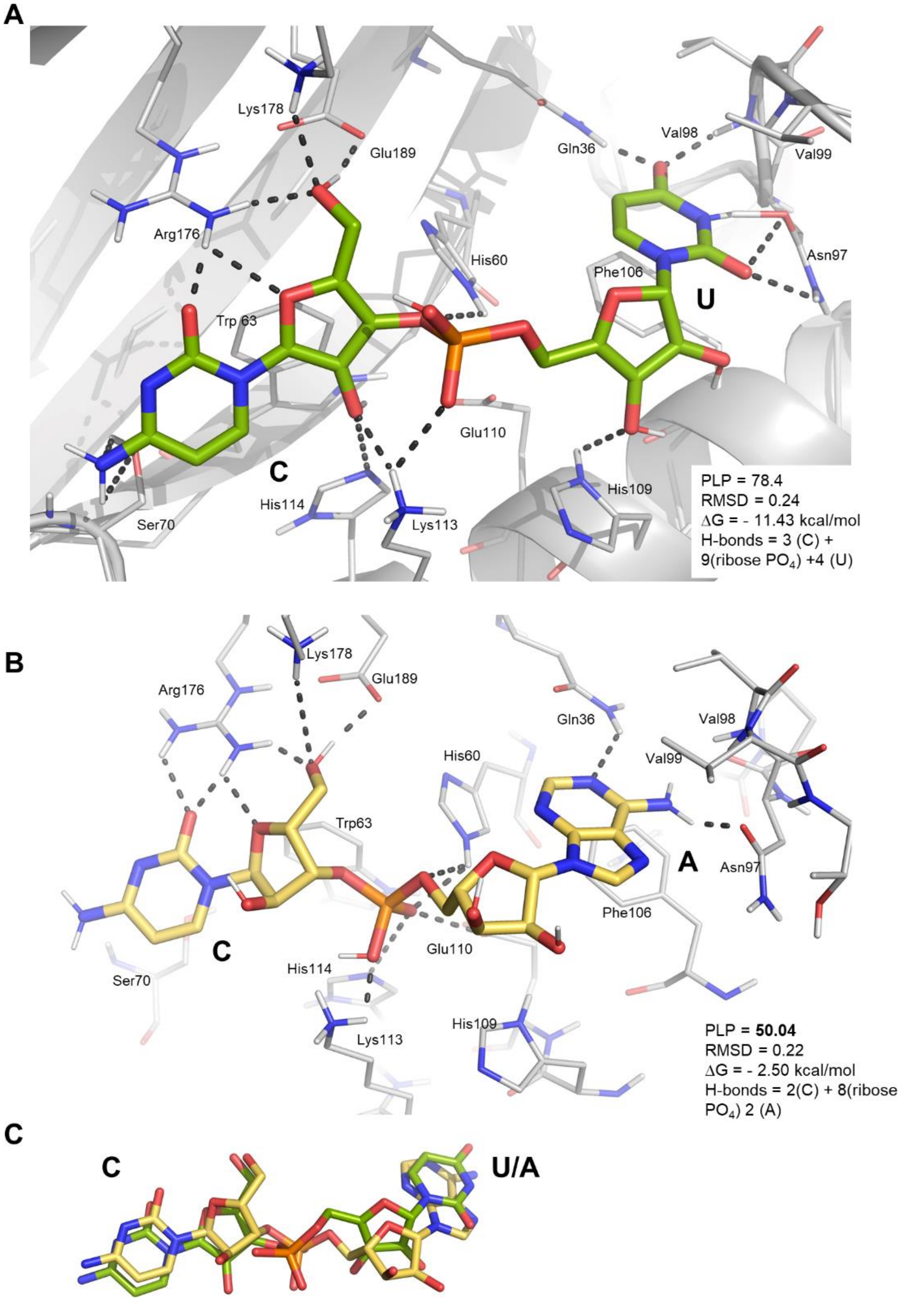

Figure S11. Molecular docking of cusativin with CpU and CpA ligands.

Homology model structure of cusativin was docked with (**A**) CpU (green) and (**B**) CpA (yellow) dinucleotide ligands. **C.** Overlay of the docked CpU and CpA ligands.

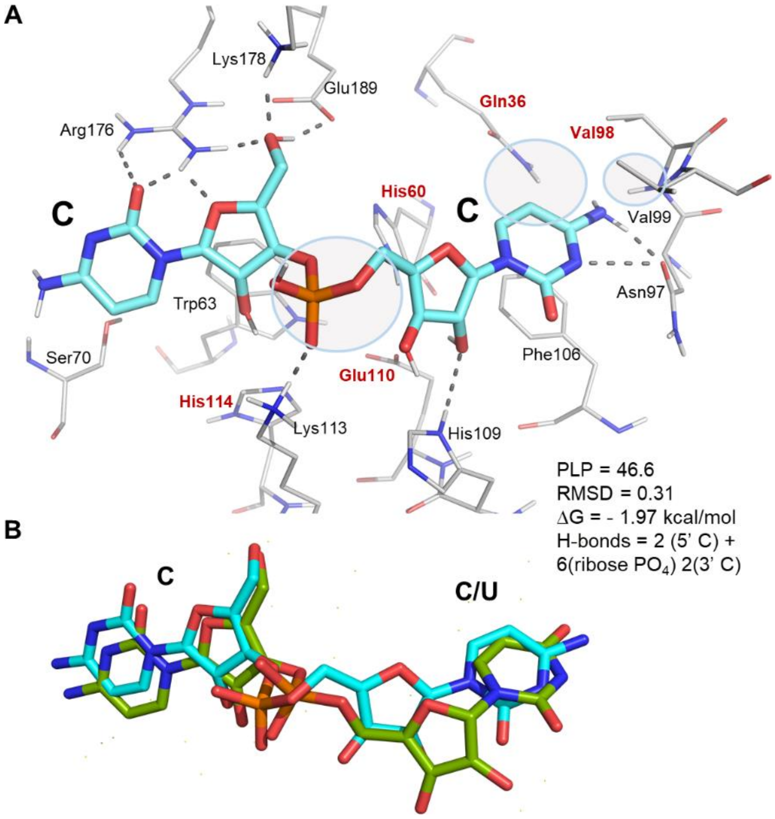

Figure S12. Molecular docking of cusativin with CpC ligand.

A. Docked status of CpC ligand in the active site of cusativin. Amino acid residues that are not making bonds with CpC ligand are shown in red font and gray color filled circles represent the regions involving missed interactions. **B.** Overlay of docked CpC (cyan) and CpU (green) ligands.

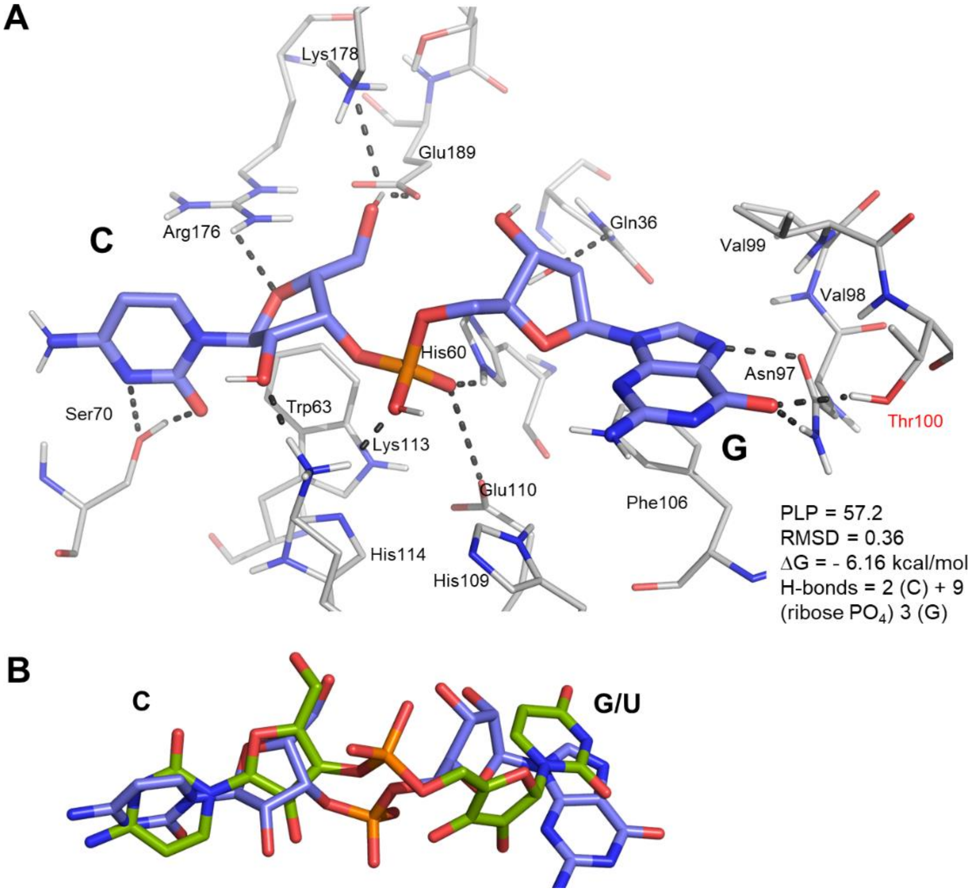

Figure S13. Molecular docking of cusativin with CpG ligand.

A. Docked status of CpG ligand in the active site of cusativin. Amino acid that is making unique interactions to the CpG ligand is shown in red font. **B.** Overlay of docked CpG (blue) and CpU (green) ligands.

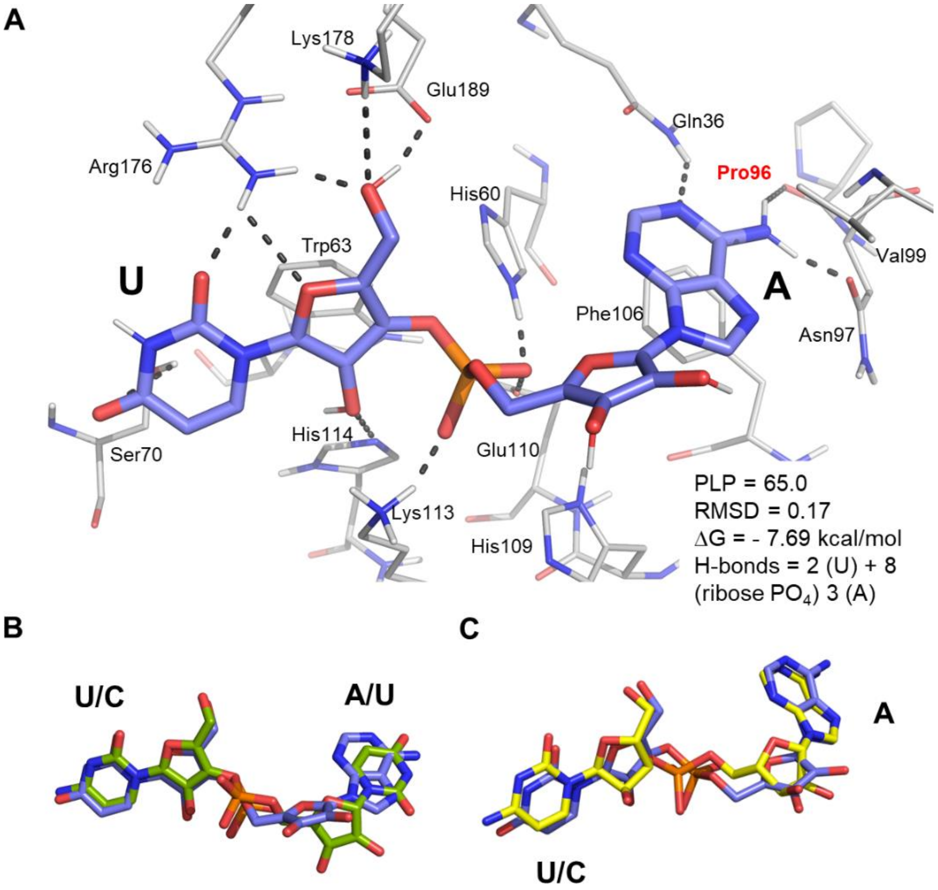

Figure S14. Molecular docking of cusativin with UpA ligand.

A. Docked status of UpA ligand in the active site of cusativin. Amino acid residue making interactions unique to the UpA ligand is shown in red font. **B.** Overlay of docked UpA (blue) and CpU (green) ligands. **C.** Overlay of docked UpA (blue) and CpA (yellow) ligands.

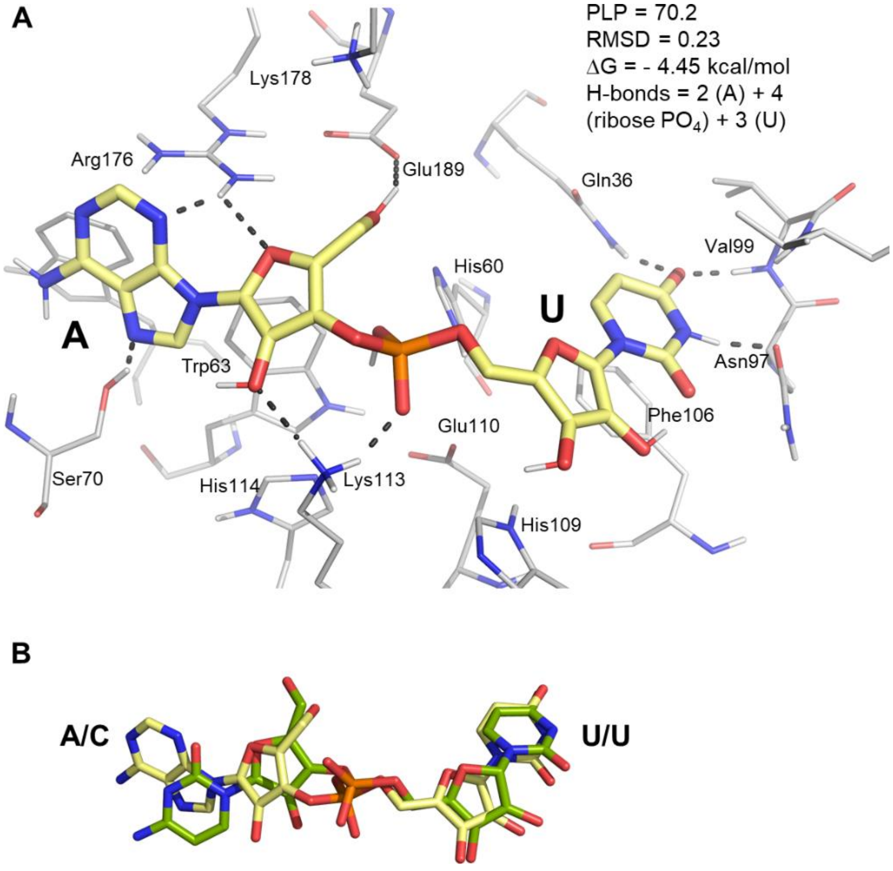

Figure S15. Molecular docking of cusativin with ApU ligand.

A. Docked status of ApU ligand in the active site of cusativin. **B.** Overlay of docked ApU (yellow) and CpU (green) ligands.

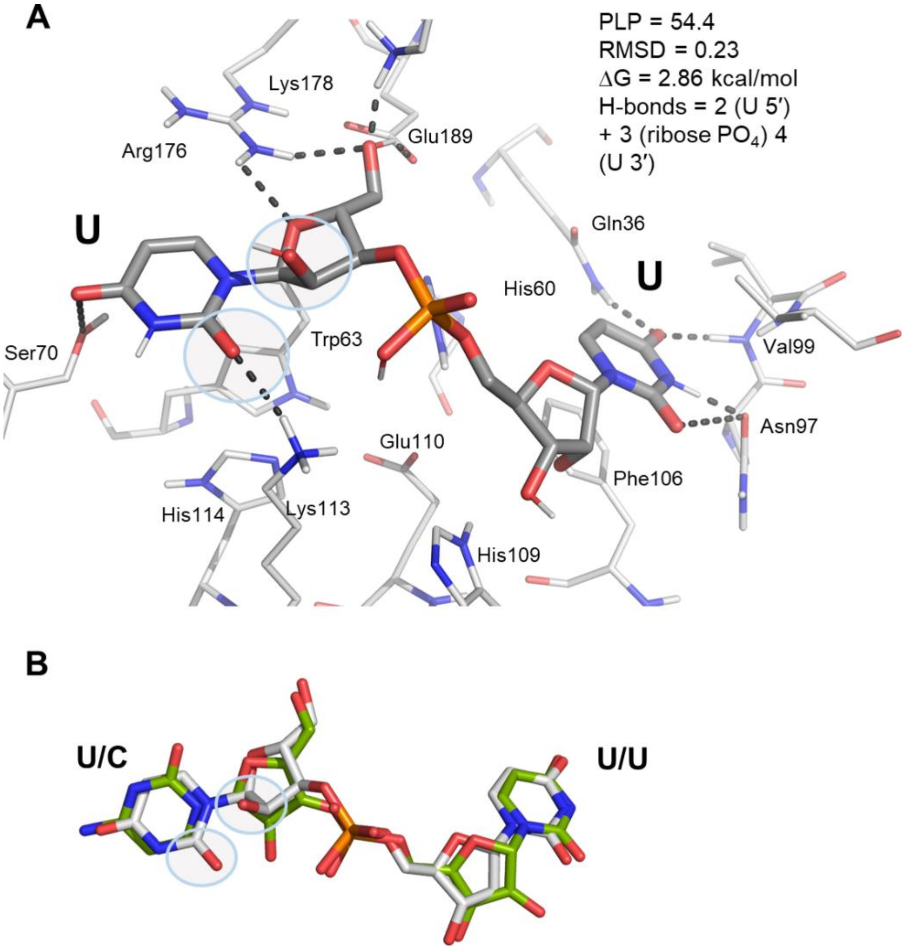

Figure S16. Molecular docking of cusativin with UpU ligand.

A. Docked status of UpU ligand in the cusativin active site. Altered orientation of the chemical groups in the ligand are denoted by grey color filled circles. **B.** Overlay of docked UpU (grey) and CpU (green) ligands.

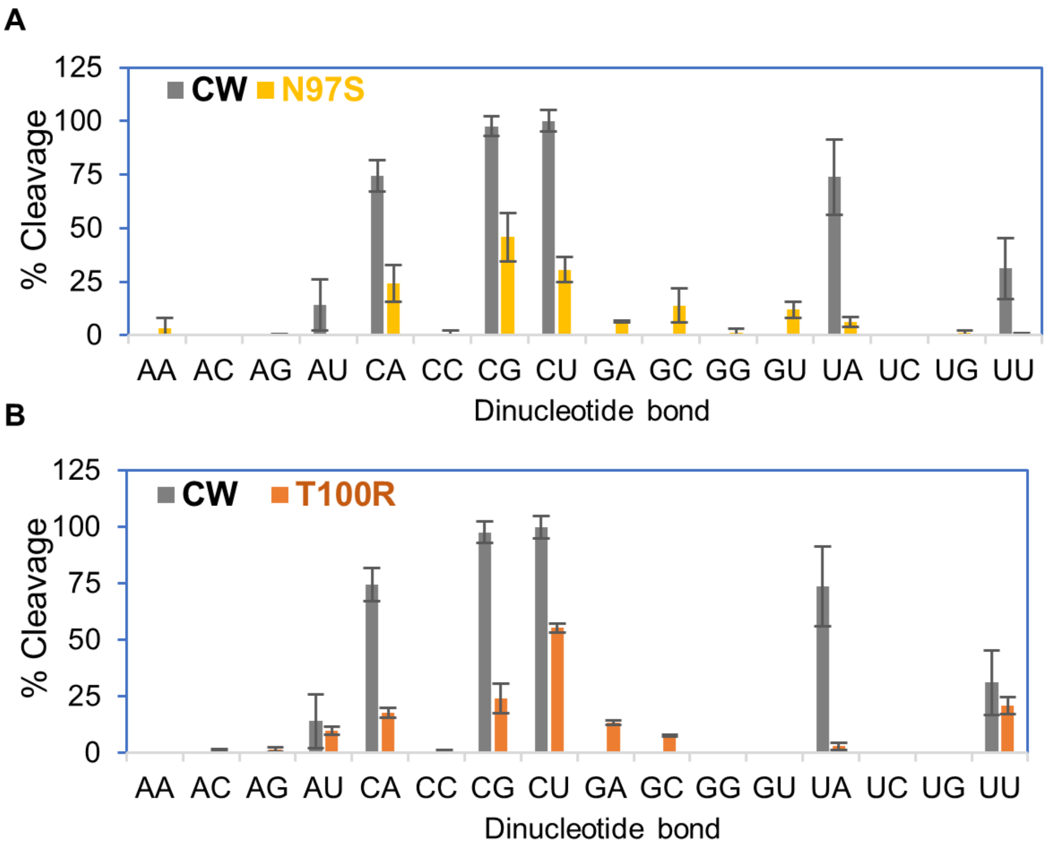

Figure S17. Altered RNA cleavage characteristics of N97S (**A**) and T100R (**B**) mutant proteins of cusativin. Dinucleotide bonds of 31-mer RNA cleaved by the RNase cusativin are indicated on the x-axis. Bars represent the cleavage percentage for wild type (CW-grey) and mutant proteins (N97S – gold, T100R – orange) for the target dinucleotide bond. Absence of bar indicate no cleavage of the bond.

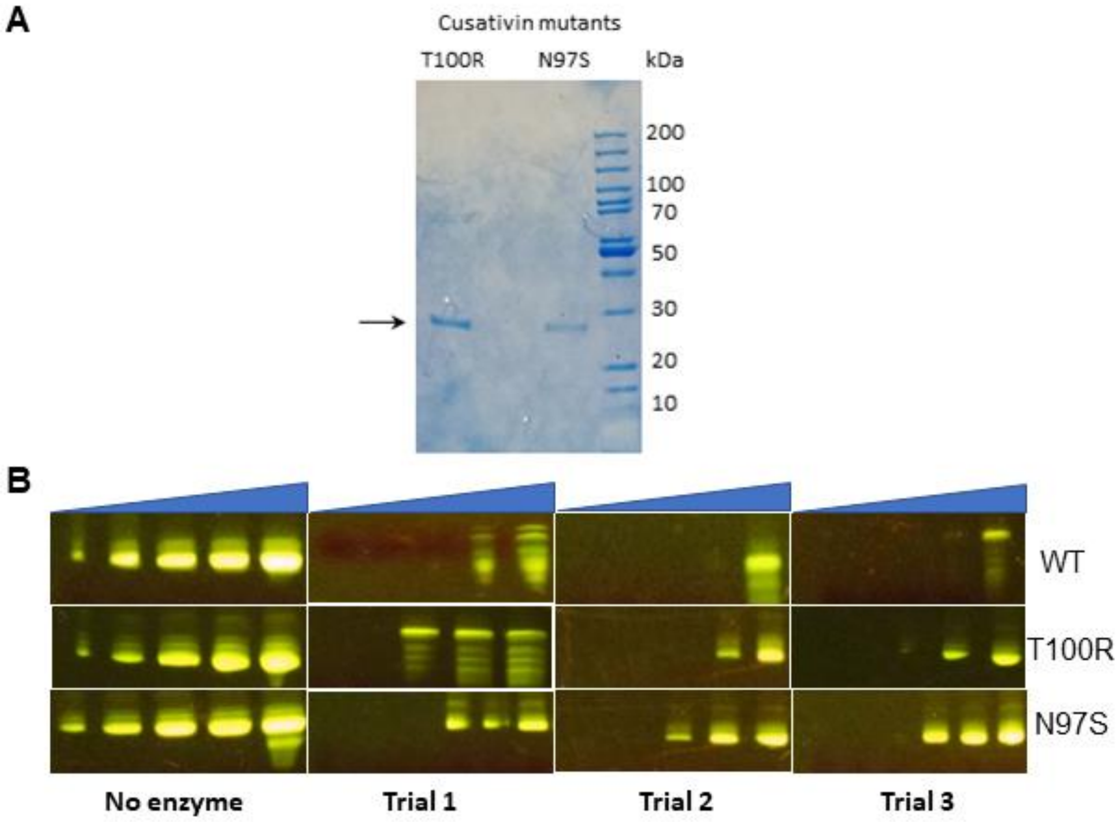

Figure S18. A. Sodium dodecyl sulfate-polyacrylamide gel electrophoresis (SDS-PAGE) of cusativin mutant proteins. Following purification on Ni^{+}-agarose beads, ~1 µg of the protein preparation was electrophoresed through a 4-20% polyacrylamide gradient gel following denaturation with Laemmli buffer and stained with Coomassie brilliant blue. Cusativin with T100R and N97S mutants are indicated on top. The position of purified polypeptide (~27 kDa) is indicated by an arrow.

B. Enzyme kinetic analysis. Increasing amounts of RNA (0.5, 1, 2, 3, and 4 µg of yeast $tRNA^{Phe}$ was treated with buffer (no enzyme) or 250 ng of cusativin variant (WT, T100R and N97S) proteins, and subjected to 15% polyacrylamide-urea gel electrophoresis. ImageJ was used to compute the change in fluorescence intensity of RNA band to calculate the kinetic data, Km, Vmax and Kcat.

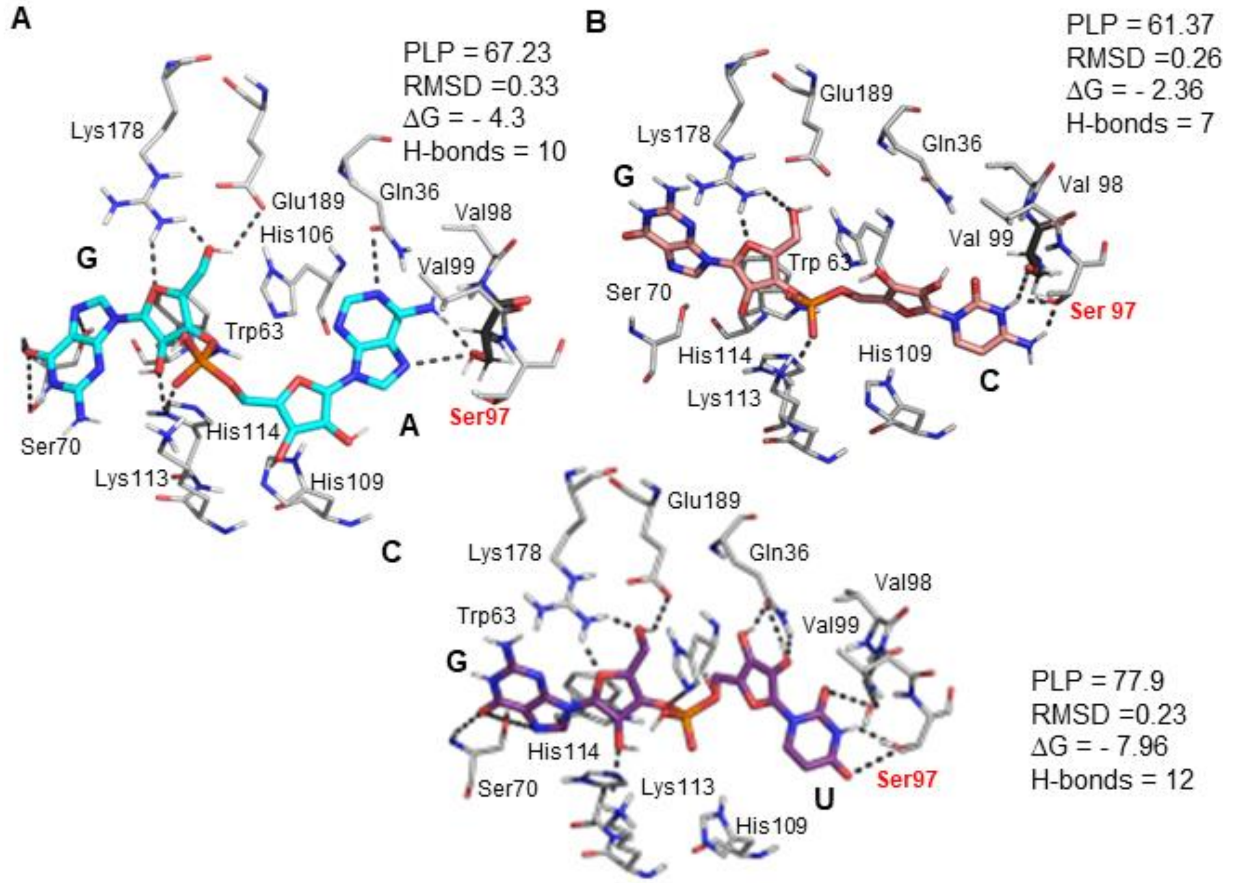

Figure S19. Cusativin N97S mt was docked with GpA **(A),** GpC **(B),** and GpU **(C)** dinucleotide ligands. The complex shows hydrogen bonding interactions with active site residues with appropriately positioned dinucleotide. The mutant residue is indicated by red font.

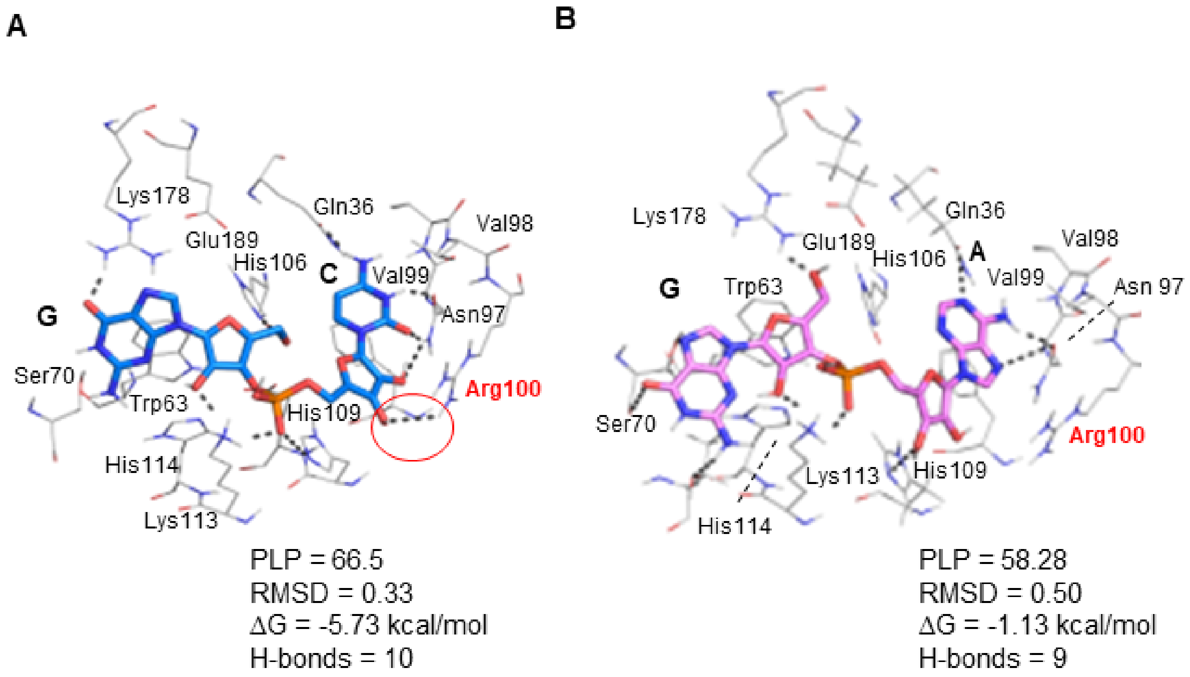

Figure S20. Cusativin T100R mt was docked with GpC **(A)** and GpA **(B)** dinucleotide ligands. The complex shows hydrogen bonding interactions with active site residues with appropriately positioned dinucleotide. The mutant residue is indicated by red font and its hydrogen bonding interaction is circled.
